# Coupling photocatalytic CO_2_ reduction and CH_3_OH oxidation for selective dimethoxymethane production

**DOI:** 10.1038/s41467-024-49927-1

**Published:** 2024-07-18

**Authors:** Yixuan Wang, Yang Liu, Lingling Wang, Silambarasan Perumal, Hongdan Wang, Hyun Ko, Chung-Li Dong, Panpan Zhang, Shuaijun Wang, Ta Thi Thuy Nga, Young Dok Kim, Yujing Ji, Shufang Zhao, Ji-Hee Kim, Dong-Yub Yee, Yosep Hwang, Jinqiang Zhang, Min Gyu Kim, Hyoyoung Lee

**Affiliations:** 1 Department of Chemistry, Sungkyunkwan University, 2066 Seobu-Ro, Suwon, 16419 Republic of Korea; 2 Creative Research Institute, Sungkyunkwan University, 2066 Seobu-Ro, Suwon, 16419 Republic of Korea; 3 CO2 to Multicarbon Production Center, Sungkyunkwan University, 2066 Seobu-Ro, Suwon, 16419 Republic of Korea; 4 Institute of Quantum Biophysics, Sungkyunkwan University, 2066 Seobu-Ro, Suwon, 16419 Republic of Korea; 5 Department of Physics, Tamkang University, New Taipei City, 25137 Taiwan; 6 School of Material Science and Engineering, Jiangsu University, Zhenjiang, 212013 People’s Republic of China; 7 School of Energy and Power Engineering, Jiangsu University, Zhenjiang, 212013 People’s Republic of China; 8 Department of Energy Science, Sungkyunkwan University, 2066 Seobu-Ro, Suwon, 16419 Republic of Korea; 9 School of Chemical Engineering, The University of Adelaide, Adelaide, SA 5005 Australia; 10 Beamline Research Division, Pohang Accelerator Laboratory, Pohang University of Science and Technology, Pohang, 37673 Republic of Korea

**Keywords:** Photocatalysis, Solar energy, Photochemistry, Materials for energy and catalysis

## Abstract

Currently, conventional dimethoxymethane synthesis methods are environmentally unfriendly. Here, we report a photo-redox catalysis system to generate dimethoxymethane using a silver and tungsten co-modified blue titanium dioxide catalyst (Ag.W-BTO) by coupling CO_2_ reduction and CH_3_OH oxidation under mild conditions. The Ag.W-BTO structure and its electron and hole transfer are comprehensively investigated by combining advanced characterizations and theoretical studies. Strikingly, Ag.W-BTO achieve a record photocatalytic activity of 5702.49 µmol g^−1^ with 92.08% dimethoxymethane selectivity in 9 h of ultraviolet-visible irradiation without sacrificial agents. Systematic isotope labeling experiments, in-situ diffuse reflectance infrared Fourier-transform analysis, and theoretical calculations reveal that the Ag and W species respectively catalyze CO_2_ conversion to *CH_2_O and CH_3_OH oxidation to *CH_3_O. Subsequently, an asymmetric carbon-oxygen coupling process between these two crucial intermediates produces dimethoxymethane. This work presents a CO_2_ photocatalytic reduction system for multi-carbon production to meet the objectives of sustainable economic development and carbon neutrality.

## Abstract (web summary)

This study unravels the efficient photocatalytic route for synthesizing dimethoxymethane by coupling CO2 reduction integrated with CH3OH oxidation by using a silver and tungsten comodified blue titanium dioxide catalyst under mild conditions.

## Introduction

**Fig. 1:**
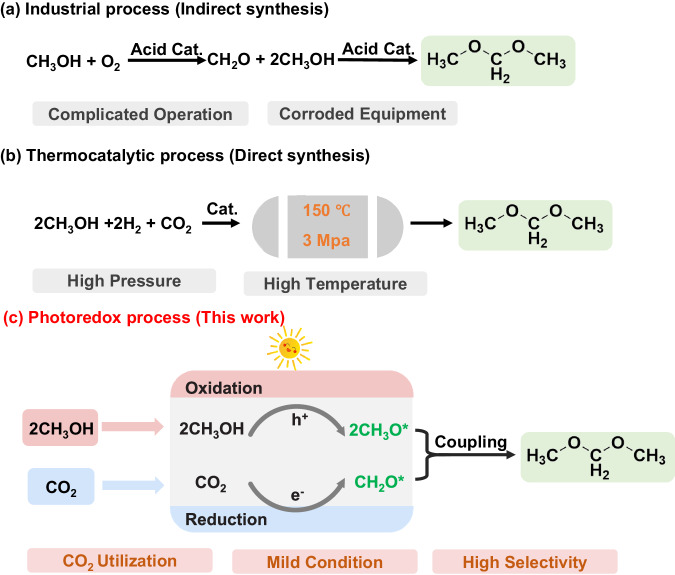
Comparison of the different DMM synthesis route. **a** Industrial (indirect) route. **b** Current thermocatalytic (direct) process. **c** Photoredox route.

Dimethoxymethane (DMM, CH_3_O-CH_2_-OCH_3_) is an attractive compound for numerous applications, including its use as a fuel additive that can enhance diesel fuel yields and as a precursor of oxymethylene dimethyl ether^1–3^. Meeting the growing demand for DMM relies in large part on an indirect synthesis route (Fig. 1a)^4,5^. Specifically, methanol (CH_3_OH) is oxidized by oxygen (O_2_) to create formaldehyde (O = CH_2_), which is then combined with other two CH_3_OH molecules to produce DMM. Despite the economic efficiency of the process, it necessitates complex operating conditions and inevitably leads to equipment corrosion due to the requirement for strongly acidic catalysts. In addition, undesirable peroxide products and carbon oxides are easily produced due to the strong oxidation ability of O_2_^6,7^. To avoid by-product generation associated with the CH_3_OH oxidation route, the CH_3_OH dehydrogenation to DMM was explored. The synthesis of DMM via dehydrogenation entails the non-oxidative conversion of methanol into the formaldehyde intermediate, followed by subsequent acetalization reaction of formaldehyde with methanol to yield DMM. For example, Palkovits et al. reached over 80% selectivity of DMM over the Cu/zeolite catalyst under a gas-phase reactor^8^. Most recently, To et al. achieved 40% of the DMM equilibrium-limited yield under mild conditions (200 °C, 1.7 atm) based on Cu-zirconia-alumina (Cu/ZrAlO) catalyst^9^. Nonetheless, the primary challenge is the requirement for harsh conditions, such as high reaction temperatures, to overcome the thermodynamic constraints of gas-phase CH_3_OH dehydrogenation.

Since carbon dioxide (CO_2_) emissions contribute to global warming^10,11^, the conversion of CO_2_ to a high-value chemical with a lower global warming potential could make a substantial contribution to the global effort to mitigate climate change^12^. Recently, CO_2_ has been considered a feedstock for DMM production through a more environmentally friendly process, one of the most popular approaches is direct synthesis by reacting CO_2_ and CH_3_OH. For example, CO_2_, a weak oxidizer, can selectively oxidize CH_3_OH to produce DMM at 150 °C and 3 Mpa (Fig. 1b)^13^. Although such strategies can alleviate environmental concerns and minimize equipment corrosion, synthetic routes require high temperatures and pressures. Alternatively, synthesizing value-added chemicals from a CO_2_ reduction reaction (CO_2_RR) using solar energy offers multiple advantages, including minimal pollution, low costs, and ease of operation^14^. In a conventional photocatalytic CO_2_RR process, CO_2_ is regularly coupled with water (H_2_O) to obtain valuable chemicals^15,16^. However, due to the high overpotential of H_2_O oxidation to O_2_ (1.23 V vs. a reversible hydrogen electrode (RHE)), fewer electrons are used, resulting in slower reaction kinetics^17^. H_2_O can acquire electrons to produce superoxide (O_2_^•−^) or hydroxyl radicals (•OH), which can compete with targeted CO_2_RRs. Electron donors such as triethanolamine and isopropyl alcohol are commonly used as sacrificial agents to capture holes and enhance CO_2_ photoreduction efficiency^18^. Drawbacks of this approach include wasted hole energy, increased system costs, and the transport of useless oxidation products. To tackle these problems, a dual-function, photo-redox system offers an appealing option for CO_2_RR^19^. Organic substrates can potentially substitute for H_2_O and hole scavengers in the generating value-added chemicals. Simultaneously, they can contribute by providing the necessary reducing equivalents in the form of protons and electrons to enhance the activation and reduction of CO_2_, thereby bolstering the stability and overall catalytic efficiency of interconnected reaction systems^17,20^. In this case, coupling photo-redox in CO_2_RR with an organic oxidation reaction (CH_3_OH oxidation reaction, MOR) to generate DMM under mild-range conditions appears to be a promising strategy. This is mainly attributed to the lower MOR potential (0.58 V vs. RHE), which results in high reaction efficiency and minimizes environmental impact^21^. Also, the catalytic efficiency of photocatalysis is not as high as that of thermal catalysis. There is still a need to explore photocatalysts with high selectivity and catalytic activity. To our knowledge, few reports of the synthesis of DMM with high selectivity using a photo-redox scheme involving CO_2_ and CH_3_OH have been published. The mechanism and key intermediates responsible for photo-redoxing this carbon-oxygen (C − O) coupling reaction process are also unknown.

Here, we propose that DMM can be produced by coupling CO_2_RR with MOR in a photo-redox system under mild-range conditions (Fig. 1c). Due to its strong ability to absorb visible light, blue titanium dioxide (BTO) derived from lithium-EDA-treated titanium dioxide (TiO_2_) was selected as a substrate^22,23^. Deposition of silver (Ag) and doping with a tungsten (W) species were introduced to the BTO (Ag.W-BTO) to create reduction and oxidation sites, respectively, for the dual-functional catalyst. As a result, the DMM yield reached 5702.49 µmol g^-1^ after 9 h ultraviolet (UV)-visible irradiation, and the DMM selectivity approached 92.08% without using sacrificial agents. The photoinduced charge transfer and potential mechanism were systematically explored by femtosecond transient absorption spectroscopy (fs-TA), in-situ diffuse reflectance infrared Fourier-transform spectroscopy (DRIFTS), isotope labeling experiments and density functional theory (DFT) calculations. The analyses showed that CH_3_OH meets photo-excited holes to produce *CH_3_O and H^+^ on W sites. At the same time, CO_2_ was reduced by a multi-step proton-coupled electron transfer (PCET) process to make *CH_2_O and these two intermediates finally produced DMM by asymmetric C − O coupling. This work provides a route for efficiently preparing DMM with high selectivity in mild-range photocatalytic redox conditions.

## Results

### Catalyst synthesis and structural characterization

**Fig. 2:**
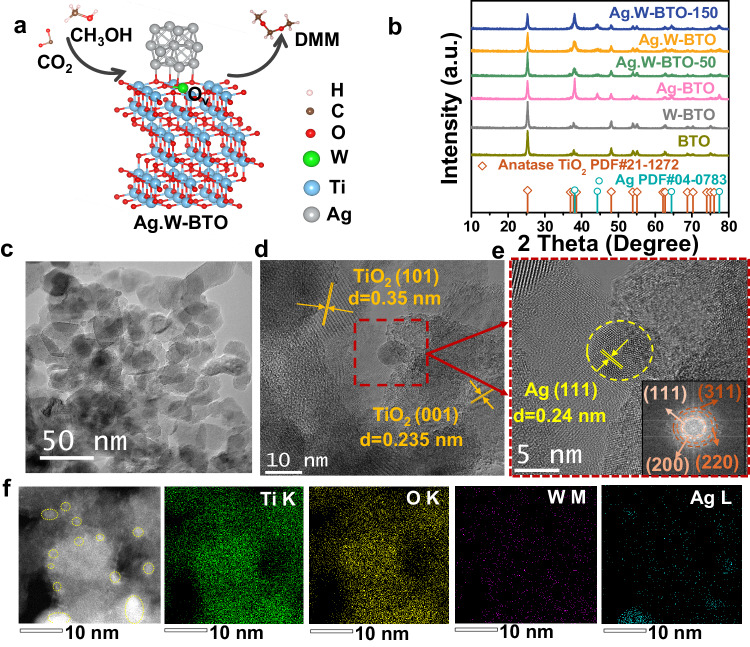
Morphology and structural characterizations of Ag.W-BTO. **a** Proposed structure of Ag.W-BTO and reaction. **b** XRD of all catalysts. **c**–**e** TEM and HRTEM images of Ag.W-BTO. **f** HAADF-STEM image of Ag.W-BTO and EDS elemental mappings of Ti, O, W, and Ag. (a. u.) represents arbitrary units.

The designed model catalysts were prepared by simply reducing WCl_6_ and AgNO_3_ with sodium borohydride (NaBH_4_) on the BTO substrate. The tungsten (W) ions were successfully introduced to the lattice of BTO, and the resulting Ag nanoparticles were in situ formed on the BTO surface during the reduction process (Fig. 2a). More details of the preparation process are supplied in the experimental section. Comparative catalysts, including BTO, Ag-BTO and W-BTO, were synthesized using the same method (Supplementary Figs. 1–11 and Supplementary Table 1). The crystal structures of all samples are shown in the X-ray diffraction (XRD) patterns of Fig. 2b. As expected, the BTO substrates in the composite samples were identified as pure anatase TiO_2_ (PDF#21-1272) after Li-EDA treatment. In this case, we expected high UV-visible light absorption and increased stability on the newly obtained BTO during the reaction^24^. The Ag nanoparticles were primarily in their metallic phase (PDF#04-0783). No prominent peak corresponding to W was evident, indicating that the W atoms were successfully doped into the BTO. The blocky Ag.W-BTO was ~40–50 nm wide, as shown in the transmission electron microscopy (TEM) image of Fig. 2c. In addition, the interplanar d spacing was 0.35 nm and 0.235 nm, shown in the Ag.W-BTO high-resolution TEM (HRTEM) image and belonging to the (101) and (001) anatase phase TiO_2_, respectively (Fig. 2d)^24^. The Ag nanoparticles were ~2–10 nm in diameter, with a clear lattice and a distance spacing of 0.24 nm, which can match Ag (111) (Fig. 2e). A fast Fourier-transform (FTT) image (inset) shows the (111), (200), (220), and (311) facets of Ag, which coincide with XRD patterns^25,26^. High-angle annular dark-field scanning TEM (HAADF-STEM) revealed Ag nanoparticles 2–10 nm in diameter (yellow circle, Fig. 2f), and energy dispersive spectrometry (EDS) was used to map the elemental distribution of Ti, O, W, and Ag. Due to the large amount of AgNO_3_ added during the preparation progress (checked by ICP-OES 38.53 wt.%; Supplementary Table 2), the Ag nanoparticles showed some aggregation of Ag.W-BTO-150 (Supplementary Fig. 5).

**Fig. 3:**
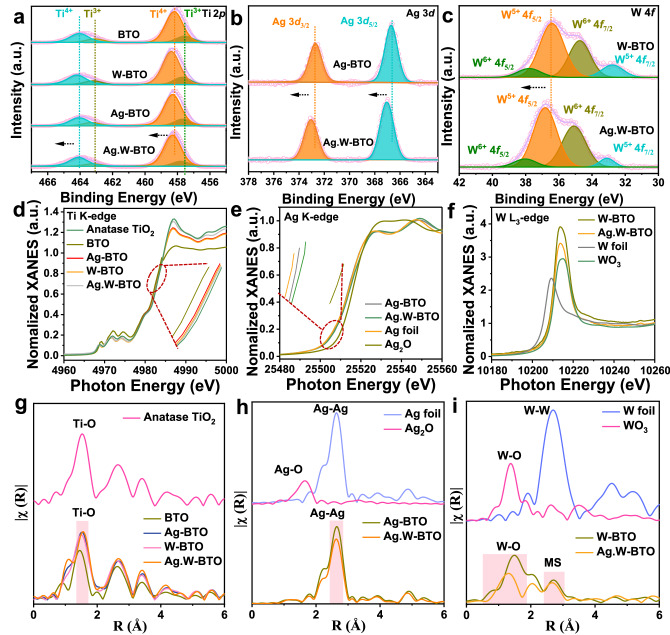
XPS and X-ray absorption fine structure (XAFS) characterizations. **a**–**c** Ti 2*p*, Ag 3*d*, and W 4 *f* XPS of BTO, W-BTO, Ag-BTO, and Ag.W-BTO, respectively. (a. u.) represents arbitrary units. **d**–**f** Normalized Ti K-edge, Ag K-edge, W L_3_-edge, and their difference X-ray absorption near edge structure (XANES) of catalysts with standard references. **g**–**i** Extended XAFS (EXAFS) k^3^ χ(k) Fourier-transform ( | X (R)|) spectra of Ti, Ag, and W in *R*-space of catalysts with standard references, respectively. R (Å) represents radial distance in Angstroms.

The chemical states of the catalysts were determined by X-ray photoelectron spectroscopy (XPS). In the Ti 2*p* spectra (Fig. 3a), four characteristic peaks appear near 464.30, 463.31, 458.64, and 457.82 eV, corresponding to Ti 2*p*_1/2_ Ti^4+^, Ti 2*p*_1/2_ Ti^3+^, Ti 2*p*_3/2_ Ti^4+^, and Ti 2*p*_3/2_ Ti^3+^, respectively^27,28^. The presence of peaks corresponding to Ti^3+^ confirms that the synthesized catalyst contains oxygen vacancies (O_v_). Compared with BTO and Ag-BTO, all W-BTO and Ag.W-BTO peaks displayed a positive shift. This phenomenon is primarily caused by W species with a lower electron cloud density and a strong electron affinity that can absorb electrons from BTO, resulting in the formation of a stable structure^29^. The Ag peaks centered at 372.60 eV and 366.70 eV can be ascribed to metallic Ag (Fig. 3b). The higher shift of the Ag binding energy in Ag.W-BTO is attributed to strong heterogeneous interaction and electron transfer between Ag and W-BTO substrate^30^. In the W 4*f* spectrum (Fig. 3c), W^6+^ 4*f*_5/2_, W^5+^ 4*f*_5/2_, W^6+^ 4*f*_7/2_, and W^5+^ 4*f*_7/2_ can be observed at 38.38, 36.72, 35.02, and 33.24 eV, respectively^31^. To prevent photo-generated carriers from recombining under light irradiation, W^6+^, as a donor just below the conduction band, can be gradually transformed to unsaturated W^5+^, which works in W^6+^/W^5+^ pairs to improve performance (Supplementary Fig. 29c)^32^. The W of Ag.W-BTO also shifted to a positive binding energy, indicating more electron transmission from W to O after Ag modification. This is attributed to the strong electron acceptor properties of O^33,34^. All the highest peaks of the O 1*s* spectra can be ascribed to Ti−O. The other peaks correspond to O_v_, an −OH/O−W group, and H_2_O (absorbed on the surface), respectively (Supplementary Fig. 12a)^35^. No boron species remained in samples during the synthesis process (Supplementary Fig. 12b). The presence of Ti^3+^ species and O_v_ were also confirmed by the Electron paramagnetic resonance (EPR) spectra (Supplementary Fig. 13). Both BTO and Ag.W-BTO show distinctive EPR signals of paramagnetic Ti^3+^ (*g* = 1.96) and O_v_ (*g* = 2.003) while TiO_2_ (P_25_) showed a negligible EPR signal^36–38^. This result implies that BTO with O_v_ was successfully synthesized after reduction with Li-EDA. The peak intensities of Ti^3+^ and O_v_ in BTO are higher than those of Ag.W-BTO, meaning the doping of W may slightly replace the Ti^3+^ or cover the O_v_ in Ag.W-BTO sample.

The electronic states of Ti, Ag, and W were explored by X-ray absorption near-edge spectroscopy (XANES). The state of Ti for all prepared samples shows features similar to those of anatase TiO_2_ (Fig. 3d). Compared with BTO, Ag-BTO, and W-BTO, the higher Ti K-edge absorption peak intensity in Ag.W-BTO is due to the collective electronic modulations of extrinsic Ag and W species. Meanwhile, the Ag in Ag-BTO and Ag.W-BTO can be traced to metallic Ag, as shown in the XRD and XPS results (Fig. 3e). However, the Ag pre-edge adsorption position of Ag.W-BTO had a positive shift compared with that of Ag-BTO, which is caused by high interaction among Ag and W-BTO support. Strong interface interactions are anticipated to promote beneficial electron transfer in the photocatalytic process. From the W L_3_-edge absorption spectra (Fig. 3f), the white line peak positions of W in W-BTO and Ag.W-BTO are higher than those of W foil and WO_3_ attributed to the strong electron adsorption properties of O in W-BTO and Ag.W-BTO, which agrees with the discussions of XPS spectra. The distinctive coordination environments of Ti, Ag, and W were investigated by Fourier-transformed (FT) extended X-ray absorption fine structure (EXAFS) spectra and fitting data (Supplementary Fig. 14 and Supplementary Tables 3−5). In the Ti FT-EXAFS spectra, Ti−O bonds can be seen at ~1.63, 1.65, and 1.62 Å for Ag-BTO, W-BTO, and Ag.W-BTO, respectively (Fig. 3g and Supplementary Fig. 14a). The Ti−O bonds of these samples are extended compared to those of pure BTO (located at ~1.52 Å) due to the introduction of W and Ag species that can stretch the Ti−O bond length and synergistically tune the local coordination environment of Ti. The dominant peaks of Ag−Ag shown in Ag-BTO and Ag.W-BTO are nearly no difference which is consistent with that of standard Ag foil (Fig. 3h and Supplementary Fig. 14b). For k^3^ χ(k)-FT of the W L_3_-edge EXAFS spectra (Fig. 3i and Supplementary Fig. 14c), the first coordination shell (W−O peak at 0.7–1.9 Å) was composed primarily of single-scattering O atoms, accompanied by multiple-scattering contributions from the second shell (peaks at 2–3 Å)^39,40^. The interatomic distances of W−O in W-BTO (~1.47 Å) and Ag.W-BTO (~1.29 Å) are different from those of standard WO_3_ (1.36 Å) as W atoms are doped into the BTO lattice rather than the isolated oxide species. Overall, the electronic interaction of Ag and W dual-active sites is expected to be capable of guaranteeing the photo-redox synergic reaction for higher efficiency.

The adsorption abilities of light in all catalysts were measured by UV-visible light spectra (Supplementary Fig. 15a)^41^. The absorption edge of Ag.W-BTO exhibited a slight redshift after W implantation and Ag deposition, which can enable stronger UV-visible light absorption and improve hot-carrier generation and catalytic performance. The band gaps of the photocatalysts were calculated using Kubelka–Munk and Tauc equations (more details are provided in the Supporting Information)^42^. The BTO and W-BTO values were 2.91 and 2.86 eV^43^, respectively (Supplementary Fig. 15b). All the BTO-related samples showed a narrower band structure than pure TiO_2_ (P_25,_ 3.2 eV), which was attributed to the existence of Ti^3+^ defect sites with multiple internal energy band gaps after Li-EDA treatment^22,44^. The valence band (VB) edge potentials and Femi levels of these catalysts were determined using Ultraviolet photoelectron spectroscopy (UPS) spectra^43^. In details, the positions of the secondary electron cutoff (*E*_cut off_) and the valence band maximum (*E*_VBM_) positions are determined using linear extrapolation of UPS^45^. As shown in Supplementary Fig. 15c, the *E*_cut off_ values of BTO and W-BTO are 17.21 and 17.49 eV, respectively, and the *E*_VBM_ values of these two samples are 1.79 and 2.15 eV, respectively. The work function (*ϕ*) can be calculated by subtracting *E*_cut off_ from the energy of the incident UV light (*hγ*) after measuring the width of the emitted electrons from the onset of the secondary electrons up to the Fermi edge, according to the Formula (1).

1 $$\phi=h\gamma - {E}_{{{{{\rm{cut\; off}}}}}}$$

Here, the energy of Helium line as a *hγ* is 21.22 eV. According to formula (1), the *ϕ* of BTO and W-BTO are calculated to be 4.01 and 3.73 eV, respectively. Consequently, the Femi levels of BTO and W-BTO are –4.01 and –3.73 eV, respectively. The VB of BTO and W-BTO in Vacuum (*E*_VAB_) were calculated using the Formula (2):

2 $${E}_{{{{{\rm{VAB}}}}}}=- ({E}_{{{{{\rm{VBM}}}}}}+\phi )\,$$

Resulting in –5.80 and –5.88 eV, respectively. All values corresponding to vacuum should be replaced with normal hydrogen electrode (NHE), resulting in a difference of –4.44 eV^46^. Therefore, band gap structures of BTO and Ag.W-BTO are shown in Supplementary Fig. 15d. The VB of W-BTO (1.44 V) exhibited higher positivity than BTO (1.36 V), imparting that doping W species theoretically facilitates MOR performance. Due that the Femi level (E_f_) of metallic Ag nanoparticles (E_f_ = –4.26 eV) is more negative than that of BTO (E_f_ = –4.01 eV) and W-BTO (E_f_ = –3.73 eV) (Supplementary Fig. 16 a, b). Take Ag.W-BTO as an example, based on the strong interfacial interaction by Mott-Schottky junction, the electrons flow from W-BTO to Ag induced by the difference in E_f_ between Ag.W-BTO until the system reaches equilibrium, resulting in band bending and Schottky barrier formation at the interface. The suitable Schottky barrier facilitates the migration of photogenerated electrons, which also proves that CO_2_RR are more likely to occur on Ag species of Ag.W-BTO during DMM synthesis process.

N_2_ adsorption/desorption analyses were carried out to determine the effect of doping W and depositing Ag on the pore structures (Supplementary Fig. 17)^47^. The presence of mesoporous structures is implied by type IV isotherms with H_3_ hysteresis loops in all prepared catalysts^48^. The pore-structure distribution curves in Supplementary Fig. 17f are evidence of the dominant mesoporous and microporous structures of all samples. The specific surface areas (SSAs) of BTO, W-BTO, Ag-BTO, and Ag.W-BTO were 28.62, 44.68, 35.26, and 52.12 m^2^ g^−1^, respectively (additional parameters are shown in Supplementary Table 6). The increased SSAs of Ag.W-BTO is likely attributed to the porous structures (Supplementary Fig. 10b).

### Photocatalytic performance by coupling CO2RR and MOR

**Fig. 4:**
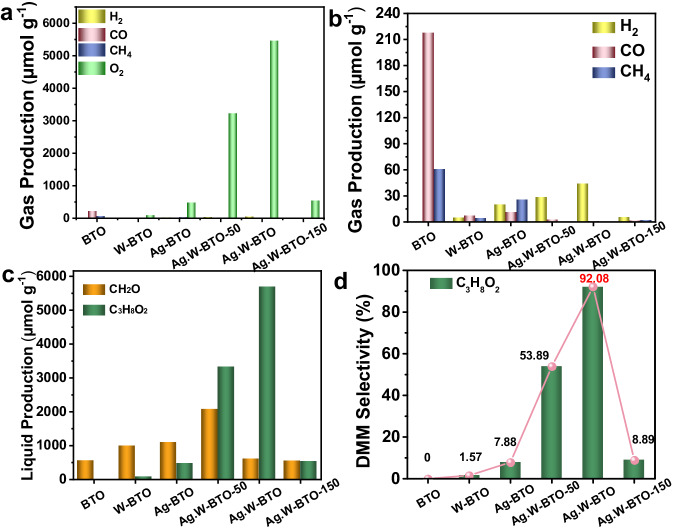
Photocatalysis CO2RR coupling with MOR performance. **a** Gas production. **b** Enlarged H_2_, CO and CH_4_ production. **c** Liquid production of prepared samples and **d** selectivity of DMM after 9 h of reaction time.

A homemade photocatalytic reaction system (Supplementary Fig. 18) was used to verify the photocatalytic performance of the samples used as photocatalysts for photo-redox CO_2_RR and MOR systems. Specifically, 30 mL of CH_3_OH was added to the reactor, which was purged with CO_2_ gas for 1 h without any use of sacrificial reagents in the dark. After irradiation with UV-visible light (320–780 nm) for 9 h, gas production (H_2_, O_2_, CO, CH_4_) and liquid production (CH_2_O and DMM) were quantitively detected by gas chromatography (GC). The corresponding productivity and selectivity of each product were calculated using Eqs. (3) – (9) as detailed in the Method section. The CO production rate on BTO reached 217.71 µmol g^-1^, which is ~100 times greater than that on Ag.W-BTO (Fig. 4a, b). In contrast, Ag.W-BTO produced the largest amount of DMM (5702.49 µmol g^-1^), but scant DMM products were seen on BTO (Fig. 4c). In addition, CH_4_, as by-product, were produced with a minimal amount of Ag.W-BTO, confirming that it is possible to make extensive use of reaction intermediates at an Ag and W dual-redox site in an Ag.W-BTO sample. The amount of CO_2_RR products (CO, CH_4_, and CH_2_O) on Ag-BTO was significantly greater than on W-BTO, which indicates that Ag prefers to be the active reduction site. Overall, W-BTO, Ag-BTO, Ag.W-BTO-50, and Ag.W-BTO-150 achieved DMM yields of 97.69, 488.47, 3337.96, and 551.11 µmol g^-1^, respectively (Fig. 4c and Supplementary Fig. 19–24). Compared with Ag-BTO and W-BTO, the greater photo-efficiency of the larger DMM yield on Ag.W-BTO was achieved because the Ag and W species individually acted as active sites to promote reductive and oxidative reactions, respectively. Ag agglomerated into large nanoparticles that covered many oxidation sites on the Ag.W-BTO-150 when excess Ag was added, resulting in less DMM and inferior selectivity (Supplementary Fig. 5). Due to producing the large amount of DMM on the Ag.W-BTO sample, the production of DMM changes was detected as time increased. As shown in Supplementary Fig. 25, the yield of DMM also exhibited a growth trend as time increased. DMM selectivity on Ag.W-BTO reached 92.08%, which is greater than that of other samples (Fig. 4d). The Apparent quantum yield (AQY) for BTO and Ag.W-BTO were measured using a range of monochromatic light band-pass filters. As depicted in Supplementary Fig. 26, the AQY of this two samples trend aligns with the UV-visible absorption spectrum, especially the AQY values of Ag.W-BTO reaching 2.15% and 1.01% at 395 nm and 420 nm, respectively. Furthermore, DMM yield and selectivity did not decrease after four catalysis cycles on Ag.W-BTO, indicating that Ag.W-BTO has excellent stability (Supplementary Fig. 27). The TEM, SEM images, XRD patterns and XPS spectrums after the cycling test were also recorded (Supplementary Fig. 28 and 29). Ag.W-BTO presents excellent sustainability of morphology and crystalline nature, which results in the higher stability of activity and selectivity for DMM synthesis. The valence states of Ti, Ag, W and O also showed nearly no difference before and after stability. The high stability is due to the fact that W is doped into the lattice of BTO with lattice distortion^49^.

Furthermore, a series of controlled experiments were performed to examine whether our photocatalytic DMM generation integrated the advantages of CO_2_RR and MOR (Supplementary Fig. 30). Ag.W-BTO at standard conditions produced DMM at a rate of 5702.49 µmol g^-1^, which is ~7 times greater than without CO_2_ (866.59 µmol g^-1^) and ~43 times greater than without CH_3_OH (132.03 µmol g^-1^). To explore the influence of pH on DMM synthesis, the pH of CH_3_OH before and after CO_2_ exposure were tested to be 6.256 and 5.202, respectively (Supplementary Fig. 31), which indicates that the introduction of CO_2_ can indeed decrease the reactant pH, which is essential for the initiation of the coupled reaction. Besides, the pH of the CH_3_OH reactant was adjusted to ~5 by using HCl (CH_3_OH + Ar, pH = 5) to avoid the CO_2_ effect. It showed that the synthesis amount of DMM in CH_3_OH + Ar with pH  = 5 condition is 533.20 µmol g^-1^ after a 9 h reaction, while that of DMM in CH_3_OH + Ar and CH_3_OH + CO_2_ without adjusting pH is about 866.59 and 5702.49 µmol g^-1^, which indicates that lower pH is not the main factor affecting the production of DMM in CH_3_OH (Supplementary Fig. 32). Moreover, to more precisely investigate the impact of *CO intermediates on the reaction, we checked DMM production by substituting CO_2_ with CO on Ag.W-BTO under the same reaction conditions. The marked increase in DMM production (7008.35 µmol g^-1^) observed when using CO and CH_3_OH as reactants, compared to the utilization of CO_2_ and CH_3_OH (5702.49 µmol g^-1^), strongly suggests the active participation of *CO intermediates in the synthesis process of DMM (Supplementary Fig. 33). Additionally, commercial TiO_2_ (P_25_) was substituted for BTO in Ag.W-BTO using the same NaBH_4_ reduction method to assess their catalytic activity. TiO_2_ was chosen for its minimal oxygen vacancies (O_v_), allowing for comparison with Ag.W-BTO to assess whether the O_v_ are active sites in the reaction. DMM yields on TiO_2_ and Ag.W-TiO_2_ were found to be ~0 and 1688.35 µmol g^-1^, respectively (Supplementary Fig. 34). The presence of DMM on Ag.W-TiO_2_ (without O_v_) suggests that O_v_ may not be the primary reactive sites. However, the lower DMM yield on Ag.W-TiO_2_ compared to Ag.W-BTO can be attributed to the weaker absorption of visible light, low specific surface area, and high electron and hole recombination rate of the TiO_2_ substrate^22,36^. Humid CO_2_ flowed instead of CO_2_ as a controlled experiment to explore the moisture effect. The produced amount of DMM is about 2307.10 µmol g^-1^. The reason why the amount decreases compared with normal conditions (CO_2_ + CH_3_OH) may be due to H_2_O oxidation in the reaction system, which competes with MOR. The high overpotential of H_2_O oxidation to O_2_ (1.23 V vs. RHE) than MOR (0.58 V vs. RHE) may result in slower reaction kinetics (Supplementary Fig. 35)^17^. In addition, when the frequency of the incident photons matches the oscillation frequency of the surface free electrons in Ag nanoparticles, the localized surface plasmon resonance (LSPR) effect occurs^50^. As for exploring the LSPR effect of deposition of Ag on the Ag.W-BTO sample, the surface temperatures of the Ag.W-BTO were assessed using an infrared thermal imager. Analysis of the temperature versus time curves for the Ag.W-BTO revealed a gradual increase from about 21–31 °C in 10 min and stabilization of about 31 °C within 30 min (Supplementary Fig. 36). This stabilization phenomenon is attributed to the equilibrium reached between the heat dissipation of the sample and its surrounding medium^46^.

**Fig. 5:**
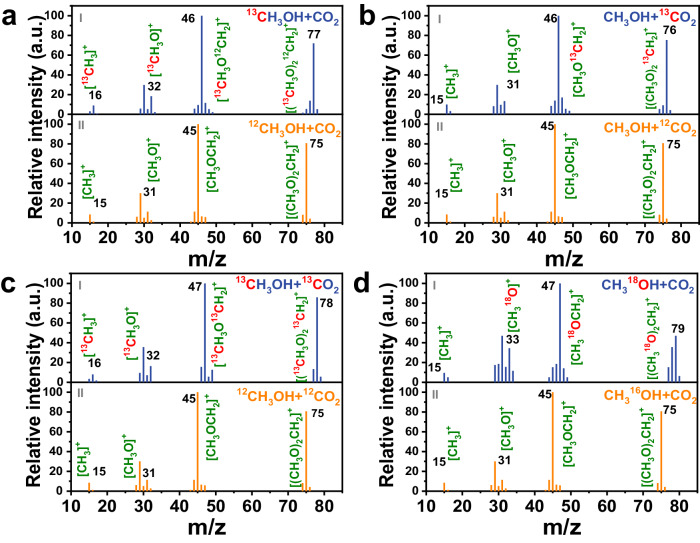
The results of isotope labeling experiments. **a** GC-MS results of I) isotope labeled ^13^CH_3_OH + CO_2_ and II) isotope non-labeled ^12^CH_3_OH + CO_2_. **b** GC-MS results of I) isotope labeled CH_3_OH + ^13^CO_2_ and II) isotope non-labeled CH_3_OH + ^12^CO_2_. **c** GC-MS results of I) isotope labeled ^13^CH_3_OH + ^13^CO_2_ and II) isotope non-labeled ^12^CH_3_OH + ^12^CO_2_. **d** GC-MS results of I) isotope labeled CH_3_^18^OH + CO_2_ and II) isotope non-labeled CH_3_^16^OH + CO_2_. (a. u.) represents arbitrary units.

To further explore the reaction mechanism and identify the carbon and oxygen sources origin of the produced DMM, the result of CO_2_ and CH_3_OH isotope-labeling experiments were determined by Gas chromatography-mass spectrometry (GC-MS). In the non-labeled DMM, the highest intensity abundance signal (base ion peak) is m/z = 45 ([CH_3_OCH_2_]^+^). The ion fragment of m/z = 75 ([CH_2_OCH_2_OCH_3_]^+^) is more stable than molecular ion fragment (m/z = 76, [CH_3_OCH_2_OCH_3_]^+^) after ionized and fragmented by a mass spectrometer. The relative abundance intensity of m/z = 75 (the second highest intensity) is much higher than m/z = 76. Therefore, the second highest intensity ion fragment of m/z = 75 can determine the DMM^51^. As depicted in Fig. 5a, the second highest intensity ion peak of DMM derived from non-labeled CH_3_OH + CO_2_ was m/z = 75 (Fig. 5a-II), while that of the DMM derived from labeled ^13^CH_3_OH + CO_2_ reached m/z = 77 (Fig. 5a-I), evidently proving that the two carbon sources in DMM originate from CH_3_OH and the one carbon originates from CO_2_. It is also observed that the m/z values of other fragments derived from ^13^CH_3_OH + CO_2_ (Fig. 5a-I) such as [^13^CH_3_]^+^ (m/z = 16) and [^13^CH_3_O]^+^ (m/z = 32), and m/z = 46 [^13^CH_3_O^12^CH_2_]^+^ are elevated by one unit higher than those of DMM derived from non-labeled CH_3_OH and CO_2_ (m/z = 15 [^12^CH_3_]^+^, m/z = 31 [^12^CH_3_O]^+^, and m/z = 45 ([^12^CH_3_O^12^CH_2_]^+^) (Fig. 5a-II), suggesting that the two terminal carbons of DMM ([^13^CH_3_O]_2_^12^CH_2_) are derived from ^13^CH_3_OH and the central carbon comes from ^12^CO_2_. In Fig. 5b, the ion fragment peak of [CH_3_]^+^ (m/z = 15) and [CH_3_O]^+^ (m/z = 31) are unchanged comparing CH_3_OH + ^13^CO_2_-derived DMM (Fig. 5b-I) and CH_3_OH + ^12^CO_2_-derived DMM (Fig. 5b-II). But, the ^13^CO_2_-derived DMM shows a base ion peak of [CH_3_O^13^CH_2_]^+^ (m/z = 46) and the secondary intensity ion fragment peak [CH_2_O^13^CH_2_OCH_3_]^+^ (m/z = 76) (Fig. 5b-I), which is only one m/z higher than that of the ^12^CO_2_ labeled case (m/z = 45 [CH_3_OCH_2_]^+^ and m/z = 75 [CH_2_OCH_2_OCH_3_]^+^) (Fig. 5b-II), which means middle position carbon in the DMM molecule originates from a CO_2_ source. In ^13^CH_3_OH + ^13^CO_2_ labeled experiments, it is clearly showed the base ion peak is m/z = 47 ([^13^CH_3_O^13^CH_2_]^+^) and the second intensity fragment is m/z = 78 ([^13^CH_2_O^13^CH_2_O^13^CH_3_]^+^) (Fig. 5c-I), respectively, which is two and three units higher than those of non-labeled m/z = 45 ([CH_3_OCH_2_]^+^) and m/z = 75 ([CH_2_OCH_2_OCH_3_]^+^) (Fig. 5c-II), respectively. In addition, comparing the result of isotope labeled ^13^CH_3_OH + ^12^CO_2_ experiments (Fig. 5a-I), the fragments of m/z = 47 ([^13^CH_3_O^13^CH_2_]^+^) and m/z = 78 ([^13^CH_2_O^13^CH_2_O^13^CH_3_]^+^) showed one unit higher than m/z = 46 ([^13^CH_3_O^12^CH_2_]^+^) and m/z = 77 ([^13^CH_2_O^12^CH_2_O^13^CH_3_]^+^), respectively. This result also confirmed that the middle carbon of DMM comes from CO_2_. Furthermore, comparing the result of isotope labeled ^12^CH_3_OH + ^13^CO_2_ experiments (Fig. 5b-I), the fragments of m/z = 47 ([^13^CH_3_O^13^CH_2_]^+^) and m/z = 78 ([^13^CH_2_O^13^CH_2_O^13^CH_3_]^+^) showed one and two unit higher than those of m/z = 46 ([^12^CH_3_O^13^CH_2_]^+^) and m/z = 76 ([^12^CH_2_O^13^CH_2_O^12^CH_3_]^+^), respectively, which exhibited that the two terminal carbons of DMM comes from CH_3_OH. In the only ^13^CH_3_OH labeled isotope experiment (Supplementary Fig. 37), the m/z = 78 peak corresponds to the ion of the [^13^CH_2_O^13^CH_2_O^13^CH_3_]^+^ structure (Supplementary Fig. 37-I), exhibiting a 3-mass unit increase compared to the non-labeled [^12^CH_2_O^12^CH_2_O^12^CH_3_]^+^ ion (Supplementary Fig. 37-II). Comparing the GC-MS results of the ^13^CH_3_OH + CO_2_ (Fig. 5a-I) and pure ^13^CH_3_OH isotope (Supplementary Fig. 37-I) experiments further confirmed not only two CH_3_OH oxidations but also CO_2_RR involvement in our DMM formation pathway. The ^18^O-labelled CH_3_^18^OH with CO_2_ isotope experiment (CH_3_^18^OH + CO_2_) was conducted to trace the O atoms in the generated DMM (Fig.5d). The base ion peak and second intensity peak of ^18^O-labled DMM are m/z = 47 ([CH_3_^18^OCH_2_]^+^) and m/z = 79 ([CH_2_^18^OCH_2_^18^OCH_3_]^+^) (Fig. 5d-I), respectively, which exhibited two and four units higher than those of non-labeled DMM (m/z = 45 [CH_3_OCH_2_]^+^ and m/z = 75 [CH_2_OCH_2_OCH_3_]^+^) (Fig. 5d-II), verifying the all the O atoms in DMM are produced by the reaction of CH_3_OH.

### Photoinduced electrons and holes separation and transportation

The separation and transportation of electrons and holes were determined by photoluminescence (PL) measurements of the BTO, W-BTO, Ag-BTO, and Ag.W-BTO catalysts (Supplementary Fig. 38)^52,53^. The PL measurements, which assess internal charge transfer in solid samples, are excited under the monochromatic wavelength (350 nm) that may not entirely mimic the actual reaction conditions. All measured samples displayed the same spectral peaks near 498 nm, while the peak intensity of Ag.W-BTO significantly declined, indicating that the Ag.W-BTO catalyst had a lower charge-carrier recombination rate. This is because Ag species tend to readily provide electrons for a CO_2_RR, while the W species prefer to provide holes to promote MOR. Ag.W-BTO displayed the strongest photocurrent response under light irradiation (Supplementary Fig. 39a). In addition, Ag.W-BTO showed the smallest arc radius of electrochemical impedance spectra (Supplementary Fig. 39b), indicating that Ag.W-BTO had the lowest charge-transfer resistance and highest photo-charge separation rate, which favor accelerated reaction kinetics and enhanced efficiency^54^.

**Fig. 6:**
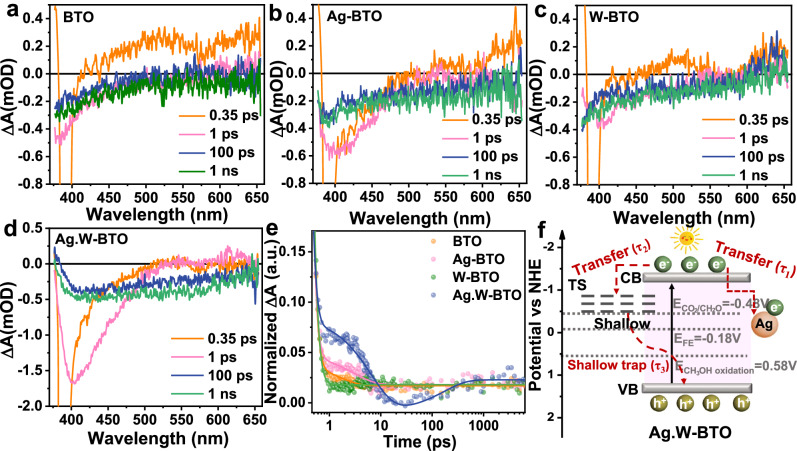
Fs-TA spectra. **a** BTO, (**b**) Ag-BTO, (**c**) W-BTO, and (**d**) Ag.W-BTO at 350 nm. **e** The normalized TA kinetics of BTO, Ag-BTO, W-BTO, and Ag.W-BTO, respectively. **f** A scheme for the charge-transfer process of Ag.W-BTO. ΔA(mOD) represents the change absorbance in milli-optical density.

Additionally, fs-TA measurements were conducted under 350 nm laser-flash photolysis to further elucidate electron transfer behavior at room temperature, with the checking conditions exhibiting disparities compared to actual reaction conditions such as temperature, pressure, and reactant composition. As depicted in Fig. 6a–d, the fs-TA spectra of BTO, Ag-BTO, W-BTO, and Ag.W-BTO included both broad negative and positive signals. The negative absorption signals can be ascribed to stimulated emission and bleaching of the ground state, and the positive absorption signals correspond to the presence of excited-state absorption by electrons^55^. The negative signals of Ag-BTO and W-BTO were stronger than those of BTO due to the instantaneous generation of charge carriers from the ground state and their direct excitation to the emission state by fast-electron transfer (Fig. 6a–c)^56,57^. As shown in Fig. 6d, the negative peak center (near 410 nm) of Ag.W-BTO was nearly three times higher than that of BTO, which can be attributed to the promotion of the utilization of electrons and holes to prevent charge carriers from recombination by Ag and W species, respectively. The dynamical decay traces and fitting of the photo-excited charge carriers for BTO, Ag-BTO, W-BTO, and Ag.W-BTO were probed at 388 nm (Fig. 6e and Supplementary Table 7), and a tri-exponential decay function was applied to fit the kinetics traces. The BTO, Ag-BTO, and W-BTO only show delay times of τ_1_ and τ_2_, which can be ascribed to electron transfer to the shallow trapping state (TS) and deep TS, respectively. However, Ag.W-BTO has three different trapping signals (τ_1_ = 0.10 ps, τ_2_ = 6.71 ps, and τ_3_ = 172.05 ps), in which τ_1_ and τ_2_ are assigned to electron transfer to a shallow Ag species and shallow TS, respectively^58^, and τ_3_ is due to the recombination of the shallow-trapped electrons with holes^59^. The final charge transfer process is shown in Fig. 6f. The electron transitions to an excited state on the conductive band (CB), leaving holes at the VB under irradiation. Subsequently, partial electrons quickly transfer to Ag sites (τ_1_) and shallow TS (τ_2_); the remaining electrons are combined with unconsumed holes (τ_3_). The longer delay time of τ_3_ indicates a lower speed for the recombination of charge electrons and holes during the photo-redox process.

### Study of reaction intermediates and mechanism

**Fig. 7:**
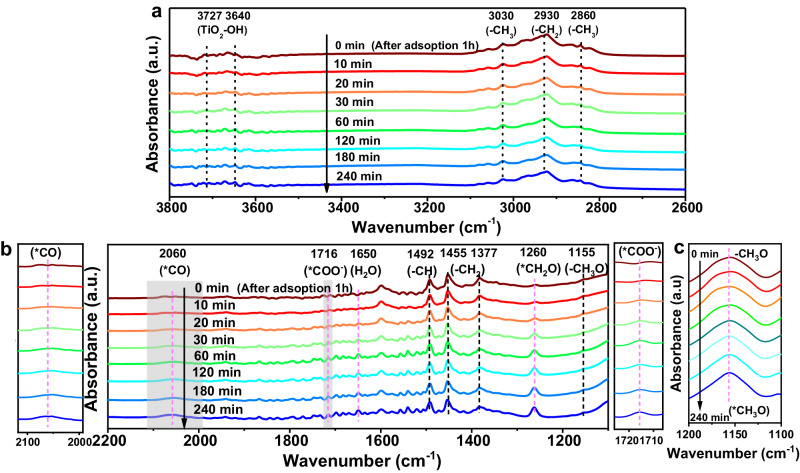
In-situ DRIFTS spectroscopy characterization of CO2 + CH3OH on Ag.W-BTO. **a**, **b** In-situ DRIFTS spectra at detailed reaction times (0, 10, 20, 30, 60, 120, 180, 240 min) with enlarged view of shaded area. **c** In-situ DRIFTS spectra from 1200 – 1100 upon doubling Ag.W-BTO amount. (a. u.) represents arbitrary units.

In-situ DRIFTS was used to identify the reaction intermediates and mechanisms in DMM synthesis. KBr served as a background (Supplementary Fig. 40) was removed before all measurements. CO_2_ and CH_3_OH were purged into the system for 1 h in the dark. All peaks were marked with dotted lines, with black cases representing negligible changes in absorption bands and colorful lines emphasizing gradual increments of the bands. Due to the continuous filling of CO_2_ gas with CH_3_OH, a high stretching vibration bond at 2300 cm^-1^ belonging to CO_2_ adsorption is evident (Supplementary Fig. 41)^59^. The band located in 1155 cm^-1^ corresponds to the spreading −CH_3_O group of CH_3_OH, which confirms that CH_3_OH is successfully adsorbed onto the Ag.W-BTO surface (Fig. 7b and c)^60^. The signals centered at 1377, 1455, 2860, and 3030 cm^-1^ were caused by the deformation of methyl groups (Fig. 7a and b**)**. The absorption bands at 1492 and 2930 cm^-1^ can be attributed to the stretching signals of C−H bonds^60,61^. The bands at 3640 and 3727 cm^-1^ correspond to the isolated −OH group (provided from CH_3_OH) and the adsorbed −OH group on the surface of TiO_2_, respectively^61^. All these bands confirm the successful absorption of CO_2_ and CH_3_OH. After UV-visible light irradiation, new intermediates emerged. Three characteristic bands appeared at 1260, 1716, and 2060 cm^-1^, corresponding to *CH_2_O, *COOH, and *CO intermediates, respectively^60,62^. Due to the small amount of sample added, the formation of the *CH_3_O intermediate for CH_3_OH as a reactant of a −CH_3_O group was not readily observed during the reaction. However, upon doubling the sample amount, a subtle increment in the −CH_3_O peak became evident as time increased, which confirmed that it produced the *CH_3_O (Fig. 7c). Furthermore, to elucidate the preference of CO_2_RR and MOR in generating *CH_2_O over *CH_3_O intermediates on Ag and W species, respectively, controlled in-situ DRIFTS experiments were conducted by flowing CO_2_ with H_2_O (CO_2_ + H_2_O) and CH_3_OH with Ar (CH_3_OH + Ar) on Ag-BTO and W-BTO catalysts, respectively. During the in-situ DRIFTS reaction of flowing CO_2_ and H_2_O on the surface of Ag-BTO, the emergence of CO_2_RR intermediates such as *CO, *COOH, and notably *CH_2_O which are located about 2060, 1716, and 1260 cm^-1^ was observed, suggesting Ag with sensitivity to CO_2_RR and its tendency to produce *CH_2_O intermediates (Supplementary Fig. 42a). Conversely, in the case of W-BTO, the production of these three intermediates, especially *CH_2_O, did not increase with reaction time, indicating a lack of sensitivity towards *CH_2_O generation (Supplementary Fig. 42b). This suggests that Ag is more inclined to produce *CH_2_O intermediates. In the in-situ DRIFTS spectrum of flowing CH_3_OH and Ar on the surface of the W-BTO catalyst, a significant increase in the peak at 1155 cm^-1^ corresponding to the *CH_3_O intermediate was observed (Supplementary Fig. 43a). However, no notable enhancement in the intensity of the *CH_3_O peak was detected on the Ag-BTO surface when CH_3_OH and Ar were introduced (Supplementary Fig. 43b). This indicates that W species are more effective in promoting the oxidation of CH_3_OH to *CH_3_O intermediates. In addition, in-situ DRIFTS experiments were conducted by flowing CO and CH_3_OH to explore whether CO participate in the reaction. The bands of *CO (2060 cm^-1^) and *CH_3_O (1155 cm^-1^) intermediates are produced immediately after the adsorption of CO and CH_3_OH for 1 h in the dark (Supplementary Fig. 44). Subsequently, a band corresponding to the *CH_2_O intermediate (around 1260 cm^-1^) reduced by *CO, appeared after UV-visible light irradiation which is consistent with our performance result. The peak intensity of these intermediates (*CO, *CH_2_O, and *CH_3_O) exhibits a smaller increase during the reaction, possibly due to the consumption of these intermediates during the synthesis of DMM at a higher reaction rate.

**Fig. 8:**
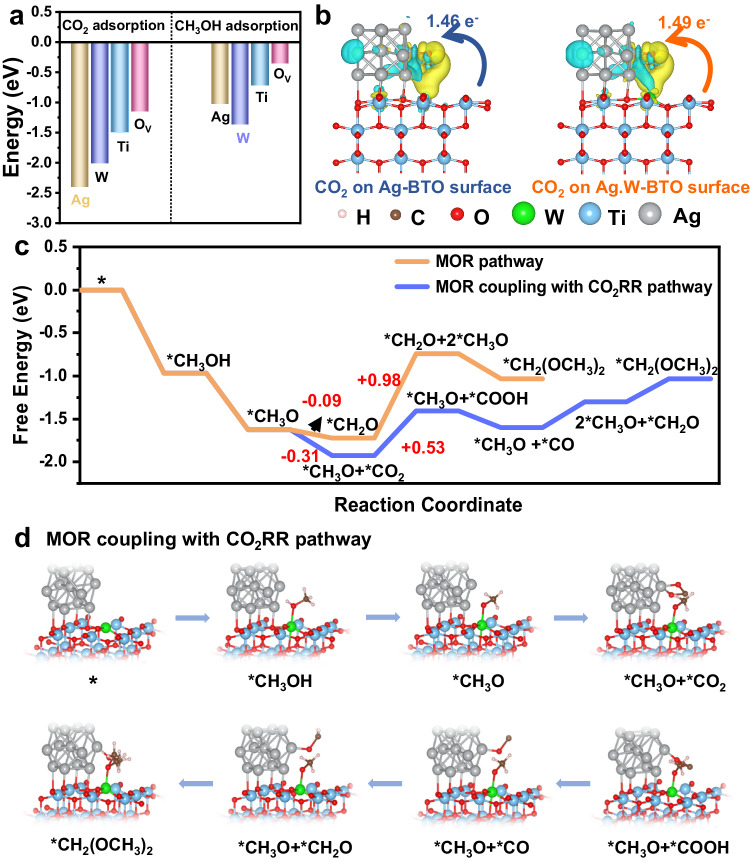
DFT calculations. **a** Calculated CO_2_ and CH_3_OH adsorption energy on the different active sites (Ag, W, Ti, and O_v_) of Ag.W-BTO, respectively. **b** Charge density difference and Bader charge analysis of CO_2_ adsorbed on the Ag-BTO and Ag.W-BTO surface. **c** Free energy diagram of DMM production via different pathway. **d** Geometries of reaction intermediates involved in MOR coupling with CO_2_RR pathways.

Although W and Ag tend to serve as oxidation and reduction sites, respectively, their roles have been confirmed by in-situ DRIFTS, band gap structures, and controlled experiments. The electronic structures and adsorption characteristics concerning CO_2_ and CH_3_OH were investigated through DFT calculations to further delve into the mechanisms and active sites for this reaction. The optimized structures of Ag.W-BTO were established based on TEM, XPS, and XANES analyses (Supplementary Fig. S45). The adsorption energies of CO_2_ and CH_3_OH on distinct active sites (W, Ag, Ti, and O_v_) were analyzed to confirm the reduction and oxidation sites on the Ag.W-BTO sample (Supplementary Fig. 46 and 47). As depicted in Fig. 8a, all the active sites (W, Ag, Ti, and O_v_) displayed negative values for CO_2_ adsorption energies, indicating a higher tendency for CO_2_ adsorption across these sites. The most negative adsorption energy for CO_2_ (–2.413 eV) signifies a preference for CO_2_ adsorption on the Ag sites within the Ag.W-BTO sample. This highlights the heightened propensity of the Ag sites within Ag.W-BTO for CO_2_RR. The most negative adsorption energy (–1.375 eV) observed for W species during CH_3_OH adsorption suggests their higher inclination towards undergoing MOR. In addition, the Charge density difference and Bader charge analyses were conducted to explore the reason for the superior performance of Ag.W-BTO. The Ag-BTO and Ag.W-BTO were chosen as an analysis model due to their better performance in CO_2_RR (Fig. 8b). The adsorbed CO_2_ species on Ag.W-BTO gained a notably higher quantity of electrons (1.49 e^-^) than on Ag-BTO (1.46 e^-^). These results confirmed more electrons can participate in the CO_2_RR process in Ag.W-BTO. It further validates that the combined effect of Ag and W facilitates the separation and transfer of electrons and holes during the reaction process on Ag.W-BTO, which is consistent with findings from fs-TA and PL data.

To further explore the formation pathway of DMM in the coexistence of CO_2_ and CH_3_OH, the free energy profile and reaction pathway of the DMM synthesis were compared on the Ag.W-BTO in different routes (MOR pathway and MOR coupling with CO_2_RR (MOR + CO_2_RR) pathway, Fig. 8c). The optimized intermediate structures corresponding to each reaction steps are displayed in Fig. 8d and Supplementary Fig. 48. The DMM formation on Ag.W-BTO through two pathways is triggered by the thermodynamical spontaneity from CH_3_OH to *CH_3_OH with an energy barrier of −0.98 eV. Then, *CH_3_OH is further oxidated to *CH_3_O with an energy barrier of −0.65 eV. Thereafter, it was found that the process from *CH_3_O to *CH_3_O + *CO_2_ with an energy barrier of –0.31 eV is more thermodynamically favorable than the oxidation of *CH_3_O to*CH_2_O with an energy barrier of −0.09 eV, which suggests that *CH_3_O tends to couple with *CO_2_ intermediates rather than to form individual *CH_2_O intermediates in CH_3_OH and CO_2_ coexist system. In addition, the process of the next step (*CH_2_O + 2*CH_3_O) in MOR pathway free energy barrier (0.98 eV) requires higher than *CH_3_O + *COOH (0.53 eV) in MOR + CO_2_RR route. That means direct oxidation of CH_3_OH to DMM is unfavorable. Moreover, three steps require energy input in the MOR + CO_2_RR pathway, including the conversion of *CO_2_ to *COOH intermediates, the reduction of *CO to *CH_2_O, and the coupling of *CH_2_O with *CH_3_O. Particularly, the conversion of CO_2_ to *COOH intermediates consumes the highest energy of 0.53 eV and is considered the rate-determining step during the DMM formation process.

According to the in-situ DRIFTS analysis, systematic isotope labeling experiments, and DFT calculations, the most probable reaction paths for the catalytic system are:

**Overall**

$${2{{{{\rm{CH}}}}}}_{3}{{{{\rm{OH}}}}}+{{{{{\rm{CO}}}}}}_{2}\to {({{{{{\rm{CH}}}}}}_{3}{{{{\rm{O}}}}})}_{2}{{{{{\rm{CH}}}}}}_{2}({{{{\rm{DMM}}}}})+{{{{{\rm{O}}}}}}_{2}$$

**In details**

$${{{{\rm{Ag}}}}}.{{{{\rm{W}}}}}\!-\!{{{{\rm{BTO}}}}}{\to }^{hv}{{{{{\rm{h}}}}}}^{+}+{{{{{\rm{e}}}}}}^{-}$$

**MOR:**

$${{{{{{\rm{CH}}}}}}_{3}{{{{\rm{OH}}}}}}\, _{{{{{\rm{ads}}}}}}\, ({{{{\rm{W}}}}})+{{{{\rm{h}}}}}^{+} \to {{\bullet }}{{{{{\rm{CH}}}}}}_{3}{{{{\rm{O}}}}}+{{{{\rm{H}}}}}^{+}$$

$${{{{{{\rm{CH}}}}}}_{3}{{{{\rm{OH}}}}}}\, _{{{{{\rm{ads}}}}}}\, ({{{{\rm{W}}}}})\to {{\bullet }}{{{{{\rm{CH}}}}}}_{3}{{{{\rm{O}}}}}+{{{{{\rm{H}}}}}}^{+}-{{{{{\rm{h}}}}}}^{+}$$

**CO**_**2**_ **RR:**

$${{{{{{\rm{CO}}}}}}_{2}}\, _{{{{{\rm{ads}}}}}}\, ({{{{\rm{Ag}}}}})+{{{{{\rm{e}}}}}}^{-}+{{{{{\rm{H}}}}}}^{+}\to {{\bullet }}{{{{\rm{COOH}}}}}$$

$${{\bullet }}{{{{\rm{COOH}}}}}+{{{{{\rm{e}}}}}}^{-}+{{{{{\rm{H}}}}}}^{+}\to {{\bullet }}{{{{\rm{CO}}}}}+{{{{{\rm{H}}}}}}_{2}{{{{\rm{O}}}}}$$

$${{{{{\rm{H}}}}}}_{2}{{{{\rm{O}}}}}\to {2{{{{\rm{H}}}}}}^{+}+{2{{{{\rm{e}}}}}}^{-}+{{\bullet }}{{{{\rm{O}}}}}$$

$${{\bullet }}{{{{\rm{CO}}}}}+{2{{{{\rm{e}}}}}}^{-}+{2{{{{\rm{H}}}}}}^{+}\to {{\bullet }}{{{{{\rm{C}}}}}{{{{\rm{H}}}}}}_{2}{{{{\rm{O}}}}}$$

**Total of CO**_**2**_ **RR**

$${{{{{{\rm{CO}}}}}}_{2}}\, _{{{{{\rm{ads}}}}}}\, ({{{{\rm{Ag}}}}})+{2{{{{\rm{e}}}}}}^{-}+{2{{{{\rm{H}}}}}}^{+}\to {{\bullet }}{{{{{\rm{CH}}}}}}_{2}{{{{\rm{O}}}}}+{{\bullet }}{{{{\rm{O}}}}}$$

$${{{{{{\rm{CO}}}}}}_{2}}\, _{{{{{\rm{ads}}}}}}\, ({{{{\rm{Ag}}}}})\to \underline{{{\bullet }}{{{{{\rm{CH}}}}}}_{2}{{{{\rm{O}}}}}+{{\bullet }}{{{{\rm{O}}}}}-{2{{{{\rm{e}}}}}}^{-}-{2{{{{\rm{H}}}}}}^{+}}$$

**Coupling process:**

$${2{{{{\rm{CH}}}}}}_{3}{{{{\rm{OH}}}}}+{{{{{\rm{CO}}}}}}_{2}\to {({{{{{\rm{CH}}}}}}_{3}{{{{\rm{O}}}}})}_{2}{{{{{\rm{CH}}}}}}_{2}+{{{{{\rm{O}}}}}}_{2}$$

$$	2(\underline{{{\bullet }}{{{{{\rm{CH}}}}}}_{3}{{{{\rm{O}}}}}+{{{{{\rm{H}}}}}}^{+}-{{{{{\rm{h}}}}}}^{+}})+(\underline{{{\bullet }}{{{{{\rm{CH}}}}}}_{2}{{{{\rm{O}}}}}+{{\bullet }}{{{{\rm{O}}}}}-{2{{{{\rm{e}}}}}}^{-}-{2{{{{\rm{H}}}}}}^{+}})\\ 	 \to (2{{\bullet }}{{{{{\rm{CH}}}}}}_{3}{{{{\rm{O}}}}}+{2{{{{\rm{H}}}}}}^{+}-{2{{{{\rm{h}}}}}}^{+})+({{\bullet }}{{{{{\rm{CH}}}}}}_{2}{{{{\rm{O}}}}}+{{\bullet }}{{{{\rm{O}}}}}-{2{{{{\rm{e}}}}}}^{-}-{2{{{{\rm{H}}}}}}^{+})\\ 	 \to 2{{\bullet }}{{{{{\rm{CH}}}}}}_{3}{{{{\rm{O}}}}}+{{\bullet }}{{{{{\rm{CH}}}}}}_{2}{{{{\rm{O}}}}}+{{\bullet }}{{{{\rm{O}}}}}+{2{{{{\rm{H}}}}}}^{+}-{2{{{{\rm{H}}}}}}^{+}\to {({{{{{\rm{CH}}}}}}_{3}{{{{\rm{O}}}}})}_{2}{{{{{\rm{CH}}}}}}_{2}({{{{\rm{DMM}}}}})+{{{{{\rm{O}}}}}}_{2}$$

CH_3_OH and CO_2_ are adsorbed on the surface of Ag.W-BTO in the dark, with more significant adsorption evidence on the W and Ag active sites, respectively. During UV-visible light irradiation, electrons (e^−^) in the VB of Ag.W-BTO are excited to the CB, while the holes (h^+^) remain in the VB. Some e^−^ transfer to Ag sites by Schottky-junction for CO_2_RR, leaving h^+^ to oxidize CH_3_OH on the W sites. CH_3_OH is oxidized to form *CH_3_O and H^+^ intermediates after binding to h^+^ on the W sites. Simultaneously, CO_2_ molecules obtain e^−^ and combine with H^+^ to produce COOH* on the active Ag sites. Furthermore, *COOH is reduced to *CO and then changed to *CH_2_O by a multi-step proton-coupled electron transfer (PCET) process. Finally, DMM is produced by coupling *CH_2_O with *CH_3_O.

## Discussion

In summary, A photo-redox system that can simultaneously couple solar-driven CO_2_RR with MOR on Ag.W-BTO dual-functional catalysts to produce a value-added chemical (DMM) was proposed. The selectivity of DMM approached 92.08% on Ag.W-BTO and was accompanied by a record-high yield of 5702.49 µmol g^-1^ after 9 h UV-visible irradiation without sacrificial agents. Validation of the synergistically coupled multi-step PCET mechanism for DMM formation was achieved through systematic isotope labeling experiments, in-situ DRIFTS analysis, and DFT calculations. The Ag species were largely responsible for the facile CO_2_ adsorption and reduction to *CH_2_O, while the W dopant promoted CH_3_OH oxidation to *CH_3_O. The two obtained intermediates couple to synthesize DMM. This work provides a concept for green photochemical synthesis of high-value chemicals by coupling CO_2_ reduction with another small molecular conversion and the fine design of photocatalysts.

## Methods

### Reagents

All chemicals were used as received without further purification. Pure titanium dioxide (TiO_2_, P_25_) was obtained from Degussa (Korea), lithium (Li), ethylenediamine (EDA), hydrochloric acid (HCl), silver nitrate (AgNO_3_), tungsten hexachloride (WCl_6_), sodium borohydride (NaBH_4_), ethanol (C_2_H_5_OH), methanol (CH_3_OH), ^13^C labeled CH_3_OH (^13^CH_3_OH), ^18^O labeled CH_3_OH (CH_3_^18^OH), formaldehyde solution (CH_2_O), and dimethoxymethane solution (DMM, C_3_H_8_O_2_) were purchased from Sigma-Aldrich (Korea). Carbon dioxide (CO_2_) gas (99.99%), hydrogen (H_2_) gas (5%), oxygen (O_2_) gas (99.99%), carbon monoxide (CO) gas (99.99%), and methane (CH_4_) gas (99.99%) were provided by Deokyang Co., LTD (Korea), ^13^CO_2_-labeled gas (99.99%, 2.16 L) was obtained by Korea noble gas Co., LTD. The ID water used in all experiments was purified using a Millipore system.

### Samples synthesis

#### Synthesis of blue TiO_2_ (BTO)

The synthesis of BTO by using the Li-treated method. Specifically, type P_25_ (1 g) and 694 mg of metallic Li were gradually put into a rubber-closed system and left for 30 min in a vacuum state. Then, 100 ml EDA was injected into this closed system under Ar gas condition with ice. These mixed reactions were stirred for 3 days. Next, 1 mol L^−1^ HCl was slowly dropped into the mixture to form Li salts and quench the excess electrons. Finally, the resulting blue material was washed with DI water several times and dried in a vacuum oven at 50 °C.

#### Synthesis of Ag.W-BTO-related samples

First, 250 mg of BTO and 500 mg of NaBH_4_ were vacuumed for 15 min, then placed in an ice bath and added to 30 mL ethanol while stirring until completely dispersed. 100 mg of WCl_6_ was dispersed in 5 mL ethanol, and 100 mg of AgNO_3_ was dispersed in 5 mL DI water, respectively. Then, slowly inject these two dispersed solutions into the BTO solution. The mixed solution was then stirred for 90 min in an ice bath before being washed many times with DI water, and ethanol and finally dried in a vacuum oven. The powder was named Ag.W-BTO. The different amounts of Ag by the same method (50 mg and 150 mg) were signaled as Ag.W-BTO-50 and Ag.W-BTO-150, respectively. W-BTO and Ag-BTO were produced using the same method except for not additionally adding the AgNO_3_ and WCl_6_.

### In-situ DRIFTS measurements

The surface of Ag.W-BTO was analyzed by in-situ DRIFTS during the CO_2_RR + MOR experiment under UV-visible light to explore the reaction mechanism. Initially, a mixture was prepared by combining 10 mg of the Ag.W-BTO sample with 190 mg of KBr powder, maintaining a weight ratio of 1:19 between the sample and KBr. The mixture was then loaded into a sample cup within the reaction cell, positioned beneath the center of three windows, comprising one quartz window and two infrared-transparent windows. Subsequently, the high-purity CO_2_ gas (99.99%) bubbling with CH_3_OH was flowed in the reaction cell for adsorbing on the catalyst for 1 h under dark conditions. Hereafter, the CO_2_ reduction reaction coupling with the CH_3_OH oxidation reaction began under the UV-visible light (320–780 nm) during continuous CO_2_ flow, and in-situ DRIFTS spectra were collected at specific reaction times. It is worth highlighting that background IR spectra were obtained by measuring KBr powder alone under identical experimental conditions. Subsequently, each background spectrum was subtracted from the corresponding DRIFTS spectrum acquired from the samples (Ag.W-BTO and KBr powder) to eliminate the influence of KBr powder in the analysis. Other reaction conditions are the same as photocatalytic reaction conditions. For checking in-situ DRIFTS spectra from 1200 to 1100 cm^-1^ on Ag.W-BTO, the process was the same as the previous method except that the Ag.W-BTO sample (20 mg), which had 10 mg of more sample, was mixed with 180 mg KBr powder (the weight ratio of the sample and KBr was 1:9).

### Photo-reaction measurements

#### Standard testing

The direct photogeneration of DMM was performed from the reaction of CO_2_ and CH_3_OH in a 50 mL stainless-steel autoclave. The system of photocatalytic experiments was set up as shown in Supplementary Fig. 18. Specifically, add 25 mg catalysts and 30 ml CH_3_OH in the stainless-steel inlet, followed by sonication for 30 min. Then, after adding the stirrer bar inside, flowing CO_2_ gas (99.99%) was used for 1 h to purge the air in the reactor in dark conditions. The autoclave was finally pressurized with CO_2_ pressure to 0.2 MPa. Finally, the reaction was performed at 373 K with stirring at 400 r.p.m. for 9 h. The light source for the photocatalysis was a 300 W Xenon lamp (320–780 nm, PLS-SXE300, Beijing Perfectlight Technology Co., Ltd). The GC system detected the amounts of products. For in-operando checking, 250 uL product was introduced to the GC at different time intervals (0, 3, 6, and 9 h). In the stability test, the CO_2_ reduction was repeated for 4 cycles under the same condition.

#### Comparison testing

For comparison, several other reaction conditions were also carried out under identical conditions to evaluate the role of the CO_2_ gas and CH_3_OH: (a) CO_2_ gas + H_2_O + Ag.W-BTO sample (Without CH_3_OH); (b) CH_3_OH + Ar + Ag.W-BTO sample (Without CO_2_); (c) CO_2_ gas + CH_3_OH + Ag.W-BTO sample under dark condition (Dark); (d) CO_2_ gas + CH_3_OH (Without catalyst). The amount of production was also checked by CG.

#### Isotopic ^13^CO_2_ experiments

^13^C-labeled CO_2_ (^13^CO_2_) gas was used as the feed gas in the labeling experiment. The reaction condition was the same as the common CO_2_ reaction conditions except for using Ar to purge the air in the reactor due to the limited supply and expense of ^13^CO_2_ gas. The final production was checked by GC-MS.

#### Isotopic CH_3_OH experiments

^13^C-labeled CH_3_OH (^13^CH_3_OH) and ^18^O-labeled CH_3_OH (^13^CH_3_OH) react with CO_2_, respectively. The final production was checked by GC-MS.

### Determination of the production

#### Quantitative test by GC

The hydrogen (H_2_) calibration data were collected and plotted. A predetermined amount of H_2_ gas (0, 0.0446, 0.0670, 0.0892, 0.111 µmol) was injected into the GC. The concentration-area curves were calibrated, and a fitted curve for which the *R*^*2*^ value was determined to be 0.999, indicating a highly strong linear correlation (Supplementary Fig. 19a). y (area) = 15114.35338x (µmol) is the linear fitting equation. After a 9 h CO_2_ reduction reaction, the produced gas (250 µL) was injected into the GC to calculate the amount.

The calibration data of carbon monoxide (CO) were obtained and plotted. Certain amounts of CO gas (0, 0.0514, 0.103, 0.154, 0.206, 0.257 µmol) were injected into GC, the concentration-area curves were calibrated, and a fitted curve exhibited the *R*^*2*^ value of 0.99907, indicating a strong liner relationship (Supplementary Fig. 19b). The linear fitting equation is y (area) = 180921.20423x (µmol). After the CO_2_ reduction reaction (9 h), the production (250 µL) gas was injected into the GC to calculate the amount.

The calibration data of methane (CH_4_) were obtained and plotted. A certain amount of CH_4_ gas (0, 0.102, 0.204, 0.306, 0.408, 0.510 µmol) was injected into GC. The concentration-area curves were calibrated, and a fitted curve for which the *R*^*2*^ value was determined to be 0.99909, indicating a highly strong linear correlation (Supplementary Fig. 19c). The linear fitting equation is y (area) = 174925.83406x (µmol). After the CO_2_ reduction reaction (9 h), the produced gas (250 µL) was injected into the GC to calculate the amount.

The calibration data of methanol (CH_3_OH) were obtained and plotted. A certain concentration of CH_3_OH liquid (4.88, 9.76, 29.28, 39.05 µmol) was injected into GC, the concentration-area curves were calibrated, and a fitted curve exhibited the *R*^*2*^ value of 0.9996 indicating a strong liner relationship (Supplementary Fig. 19d). The linear fitting equation is y (area) = 34812.349x (µmol). Before and after the CO_2_ reduction reaction (9 h), the liquid (5 µL) was injected into the GC to calculate the amount.

The calibration data of oxygen (O_2_) were obtained and plotted. A certain amount of O_2_ gas (0, 0.438, 0.875, 1.313, 1.750, 2.188 µmol) was injected into GC. The concentration-area curves were calibrated, and a fitted curve for which the *R*^*2*^ value was determined to be 0.999, indicating a highly strong linear correlation (Supplementary Fig. 19e). The linear fitting equation is y (area) = 1097.052x (µmol). After the CO_2_ reduction reaction (9 h), the produced gas (250 µL) was injected into the GC to calculate the amount.

The calibration data of formaldehyde (CH_2_O) were obtained and plotted. Certain amounts of CH_2_O were dissolved in DI water to obtain a series of concentrated solutions (0.3125, 0.625, 1.25, 2.5, 5 mmol L^-1^). These different concentration solutions (5 uL) were injected into GC, and the rotation time of CH_2_O was about 14.31 min, resulting in a calibration curve. The concentration-area curves were calibrated, and a fitted curve exhibited the *R*^*2*^ value of 0.99917, indicating a strong liner relationship (Supplementary Fig. 20). The linear fitting equation is y(area) = 659.14525x (mmol L^-1^). After the CO_2_ reduction reaction (9 h), the production (5 uL) liquid was injected into the GC to calculate the amount.

The calibration data of dimethoxymethane (C_3_H_8_O_2_) were obtained and plotted. A certain amount of C_3_H_8_O_2_ was dissolved in DI water to obtain a series of concentrated solutions (0.02, 0.04, 0.08, 0.125, 0.25, 0.5, 1 mol L^-1^). These different concentration solutions (5 uL) were injected into GC, and the rotation time of C_3_H_8_O_2_ was about 22.89 min, resulting in a calibration curve. The concentration-area curves were calibrated, and a fitted curve exhibited the *R*^*2*^ value of 0.99984, indicating a strong liner relationship (Supplementary Fig. 21). The linear fitting equation is y(area) = 2.45663x (mol L^-1^). After the CO_2_ reduction reaction (9 h), the production (5 uL) gas was injected into the GC to calculate the amount.

#### Qualitative analysis by GC-MS

After the CO_2_ reduction reaction, the production was injected into GC-MS (5 µL).

### Calculation of Selectivity

The gas products (H_2_, CO, CH_4_ and O_2_) amount (μmol g^-1^), liquid products (CH_2_O, C_3_H_8_O_2_) amount (μmol g^-1^) after 9 h reaction were calculated using equations presented in the following:

3 $${{{{{\rm{H}}}}}}_{2}=\frac{{Vr}*S}{15114.35338*W*{Vn}*1000}$$

4 $${{{{\rm{CO}}}}}=\frac{{Vr}*S}{180921.20423*W*{Vn}*1000}$$

5 $${{{{{\rm{CH}}}}}}_{4}=\frac{{Vr}*S}{174925.83406*W*{Vn}*1000}$$

6 $$\,{{{{{\rm{O}}}}}}_{2}=\frac{{Vr}*S}{1097.052*W*{Vn}*1000}$$

7 $${{{{{\rm{CH}}}}}}_{2}{{{{\rm{O}}}}}=\frac{{VL}*S}{659.14525*1000*W}*1000$$

8 $${{{{{\rm{C}}}}}}_{3}{{{{{\rm{H}}}}}}_{8}{{{{{\rm{O}}}}}}_{2}=\frac{{VL}*S}{2.45663*{10}^{6}*W}*1000$$

9 $${{{{\rm{Selectivity}}}}}={{{{\rm{Yield}}}}}\;{{{{\rm{DMM}}}}}*2/{{{{\rm{Consumption}}}}}\;{{{{{\rm{CH}}}}}_{3}{{{{\rm{OH}}}}}}*100\%$$

Where *Vr* (mL) is the volume of the reactor, *VL* (mL) is the volume of liquid, *Vn* (μL) represents product volume injected into GC, *S* is the area of the product obtained by GC, and *W* (g) is the mass of catalyst.

### Supplementary information

Supplementary Information

Peer Review File

### Source data

Source data

## Data Availability

The data that support the conclusions of this study are available within the paper and supplementary information. Source data are provided with this paper. Figshare 10.6084/m9.figshare.25713021. Source data are provided with this paper.
