# Supplementary material for: Coupling photocatalytic CO2 reduction and CH3OH oxidation for selective dimethoxymethane production
Source: Nat Commun. 2024 Jul 18;15:6047. doi: 10.1038/s41467-024-49927-1 (PMC11258228; doi:10.1038/s41467-024-49927-1)
Supplement: Supplementary file 1 — Supplementary Information [file 41467_2024_49927_MOESM1_ESM.pdf]

Coupling photocatalytic CO_2 reduction and CH_3OH oxidation for selective dimethoxymethane production

Yixuan Wang[1,2,3#], Yang Liu[1,2#], Lingling Wang[1,2], Silambarasan Perumal[1,2,3], Hongdan Wang[1,2], Hyun Ko[4], Chung-Li Dong[5], Panpan Zhang[6], Shuaijun Wang[7], Ta Thi Thuy Nga[5], Young Dok Kim[1], Yujing Ji[1], Shufang Zhao[1], Ji-Hee Kim[8], Dong-Yub Yee[8], Yosep Hwang[1,2], Jinqiang Zhang[9], Min Gyu Kim[10] & Hyoyoung Lee[1,2,3,4]*

[1]Department of Chemistry, Sungkyunkwan University, 2066 Seobu-Ro, Suwon, 16419, Republic of Korea

[2]Creative Research Institute, Sungkyunkwan University, 2066 Seobu-Ro, Suwon, 16419, Republic of Korea

[3]CO_2 to Multicarbon Production Center, Sungkyunkwan University, 2066 Seobu-Ro, Suwon, 16419, Republic of Korea

[4]Institute of Quantum Biophysics, Sungkyunkwan University, 2066 Seobu-Ro, Suwon, 16419, Republic of Korea

[5]Department of Physics, Tamkang University, New Taipei City, 25137, Taiwan

[6]School of Material Science and Engineering, Jiangsu University, Zhenjiang 212013, People's Republic of China

[7]School of Energy and Power Engineering, Jiangsu University, Zhenjiang 212013, People's Republic of China

[8]Department of Energy Science, Sungkyunkwan University, 2066 Seobu-Ro, Suwon, 16419, Republic of Korea

[9]School of Chemical Engineering, The University of Adelaide, Adelaide, SA, 5005, Australia

[10]Beamline Research Division, Pohang Accelerator Laboratory, Pohang University of Science and Technology, Pohang, 37673, Republic of Korea

[#] These authors contributed equally: Yixuan Wang, Yang Liu

*e-mail: hyoyoung@skku.edu

Material Characterizations

The samples' morphology was investigated using a JSM 7401F (JEOL Ltd., Tokyo, Japan) scanning electron microscope (SEM) operated at 3.0 kV. High-resolution TEM (HRTEM) images and High-angle annular dark-field scanning transmission electron microscopy (HAADF-STEM) and EDS elemental mapping images were collected on a JEOL ARM-200F field-emission transmission electron microscope operating at a 200 kV accelerating voltage. X-ray photoelectron spectroscopy (XPS), which is located at the chiral material core facility center of Sungkyunkwan University, was performed using a VG Microtech ESCA 2000 equipped with a monochromic Al X-ray source (97.9 W, 93.9 eV). X-ray diffraction (XRD) patterns were acquired using a Rigaku Ultima IV, using the substrates directly. The Inductively Coupled Plasma Optical Emission Spectroscopy (ICP-OES) was checked by Agilent 5100/PerkinElmer AVIO 550Max. The In-situ diffuse reflectance infrared Fourier-transform spectra (DRIFTS) were immediately obtained by an FT-IR spectrometer (Thermo-Fisher Scientific, Nicolet iS10, USA), which is located at the Chiral Material Core Facility Center of Sungkyunkwan University, with a reaction system consisting of a praying mantis DRIFTS accessory (Harrick Scientific, USA) and a reaction cell (HVC, Harrick Scientific, USA). The X-ray absorption spectroscopy (XAS) measurements were performed at the BL10C beamline of the Pohang Light Source (PLS-II, Korea). Athena and Artemis software analyzed the acquired XAFS data according to the

standard process. The spectra were calibrated, averaged, pre-edge background subtracted, and post-edge normalized using the Athena program. The Fourier transformation of EXAFS oscillations from k to R space was achieved to obtain a radial distribution function. All data fitting was completed in the Artemis program. The 300 W Xenon lamp (320-780 nm, PLS-SXE300, Beijing Perfectlight Technology Co., Ltd) provided the UV-visible light source. The BET (Brunauer–Emmett–Teller) and HK (Horvath–Kawazoe) methods were used to determine the specific surface area and micropore analysis, respectively, by using BELSORP-max through liquid N_2 cryo-sorption (Micrometrics ASAP2020, USA). Photoluminescence (PL) spectra were obtained using an Agilent Cary Eclipse Fluorescence spectrometer. The UV-Vis-diffuse reflectance spectra (DRS) were recorded on the Agilent Cary 7000. The electrochemical photo-current and impedance (EIS) were obtained using an electrochemical workstation (CHI-660E, USA) with a three-electrode system. For working electrode preparation, 5 mg of catalyst and 20 μL of Nafion solution (5%) were dispersed into a 230 μL mixture solution including DI water (100 uL) and IPA (isopropanol, 130 uL) by sonication for 30 min. 250 μL of suspension was dropped onto the conductive side of FTO glass with the size of $1{\times}1cm^2$. After the sample is thoroughly dried at room temperature on the FTO glass, the mass loading of all catalysts is determined as 5 mg cm^{-2}. The Ag/AgCl electrode and Pt mash were used as the reference electrode, and counter electrode, respectively. The 15 mL 0.1 M Na_2SO_4 with 5 mL CH_3OH aqueous solution is the electrolyte. Before the electrochemical test, the CO_2 gas flowed in the electrolyte for 30 min then kept the system closed. The productions after the CO_2 reduction reaction were detected by the GC system (7890A, Agilent Technologies, USA) with porapak N and molecular sieve column. ^{13}C labeling experiments were measured by Gas Chromatograph Mass Spectrometer (GC-MS) system (YL 6900, YL instrument CO., LTD. Korea) by column–

Agilent PoraPLOT Q under temp. 150 °C 35 °C/min. The femtosecond transient absorption spectroscopy (fs-TA) was measured using a TR spectrometer (Helios, Ultrafast Systems) operating at a center wavelength of 350 nm with a bandpass filter, one operated by an optical parametric amplifier (TOPAS Prime, Light Conversion), which was used as the pump beam. The wavelength of the probe is 370-640 nm, and the pump power is about 200 nJ. The surface temperatures of the Ag.W-BTO were assessed using an infrared thermal imager (Teledyne FLIR, FLIR TG165). 25 mg of photocatalyst was positioned at a distance of 40 cm from the 300 W Xenon lamp cap (wavelength, 320-780 nm).

Band gap determination from UV-vis diffuse reflectance spectra

The UV-vis diffuse reflectance spectra were converted to absorption spectra using the Kubelka-Munk equation (1) first,

$$F(R)=\frac{(1-R)^2}{2R} \tag{1}$$

where *F(R)* and *R* represent the absorption coefficient and the relative reflectance of samples with infinite thickness in comparison to the reference, respectively. Furthermore, the band gaps of samples were estimated using the Tauc equation (2),

$$F(R)hv=A(hv-E_g)^{\frac{n}{2}} \tag{2}$$

in which *h*, *v*, *A*, and *Eg* represent the Planck constant, light frequency, proportionality constant, and optical band gap, respectively, while *n* is determined by the nature of the transition in a semiconductor. Values of 1, 3, 4, and 6 for *n* correspond to the allowed direct, forbidden direct, allowed indirect, and forbidden indirect transitions, respectively. The values of *Eg* were calculated from the plot of $(F(R)hv)^{2/n}$ against *hv* and corresponded to the intercept of the

extrapolated linear portion of the plot near the band edge with the *hv* axis. BTO-related samples were treated as semiconductors with allowed indirect transition [1].

Apparent quantum yield (AQY) calculation method

In terms of AQY, the Xe lamp was also replaced by the monochromatic LED light (CEL-LEDS35, Beijing Perfectlight Technology Co., Ltd, the wavelengths (λ) are 395, 420, 500, and 595 nm, respectively). Other experimental parameters are the same as the photocatalytic DMM production process. The light intensity was monitored by an optical power meter (CEL−NP2000−2, Beijing Perfectlight Technology Co., Ltd). The number of incident photons (*N*) is calculated by equation (3), and AQY is then calculated in equation (4).

$$N=\frac{E\lambda}{hc}=\frac{I\times S\times t\times \lambda}{hc} \quad (3)$$

$$\mathrm{AQY}=\frac{\text{the number of reacted electrons}}{\text{the number of incident photons}}\times 100\%$$

$$=\frac{[\mathrm{CO}]_n\times 2+[\mathrm{CH_4}]_n\times 8+[\mathrm{CH_2O}]_n\times 4+[\mathrm{C_3H_8O_2}]_n\times 6}{\mathrm{N}}\times 100\%$$

$$=\frac{\begin{array}{c}2\times 6.02\times 10^{23}\times \mathrm{n\ (CO)}+8\times 6.02\times 10^{23}\times \mathrm{n\ (CH_4)}+4\times 6.02\times 10^{23}\times \mathrm{n\ (CH_2O)}\\ +\,6\times 6.02\times 10^{23}\times \mathrm{n\ (C_3H_8O_2)}\end{array}}{\mathrm{N}}\times 100\% \quad (4)$$

In which *I* was the light intensity ($W\cdot m^{-2}$), *S* was the irradiation area (m^{-2}), *t* was the DMM production time (s), *λ* was the wavelength of monochromatic LED light (395, 420, 500, and 595 nm), *h* was Planck's constant (6.63×10^{-34} J·s), and *c* was the speed of light (3.8×10^{8} $m\cdot s^{-1}$). $[CO]_n$, $[CH_4]_n$, $[CH_2O]_n$ and $[C_3H_8O_2]_n$ are the number of evolved CO, CH_4, CH_2O and $C_3H_8O_2$ molecules, respectively.

DFT calculation method

All calculations were implemented using the Vienna Ab initio Simulation Package (VASP), based on DFT. We have employed the first principles[2, 3] to perform DFT calculations within the generalized gradient approximation (GGA) using the Perdew-Burke-Ernzerhof (PBE)[4] formulation. We have chosen the projected augmented wave (PAW) potentials[5, 6] to describe the ionic cores and take valence electrons into account using a plane wave basis set with a kinetic energy cutoff of 520 eV. The GGA + U method was adopted in our calculations. The value of the effective Hubbard U was set as 4.814 eV for Ti. Partial occupancies of the Kohn−Sham orbitals were allowed using the Gaussian smearing method and a width of 0.05 eV. The electronic energy was considered self-consistent when the energy change was less than 10^{-5} eV. A geometry optimization was considered convergent when the energy change was smaller than 0.05 eV $Å^{-1}$. In our structure, the U correction is used for Ni atoms. The Brillouin zone integration is performed using 2×2×1 Monkhorst-Pack k-point sampling for a structure. Finally, the adsorption energies (E_{ads}) were calculated as:

$$E_{ads}= E_{ad/sub} - E_{ad} - E_{sub} \quad (5)$$

where $E_{ad/sub}$, E_{ad}, and E_{sub} are the total energies of the optimized adsorbate/substrate system, the adsorbate in the structure, and the clean substrate, respectively.

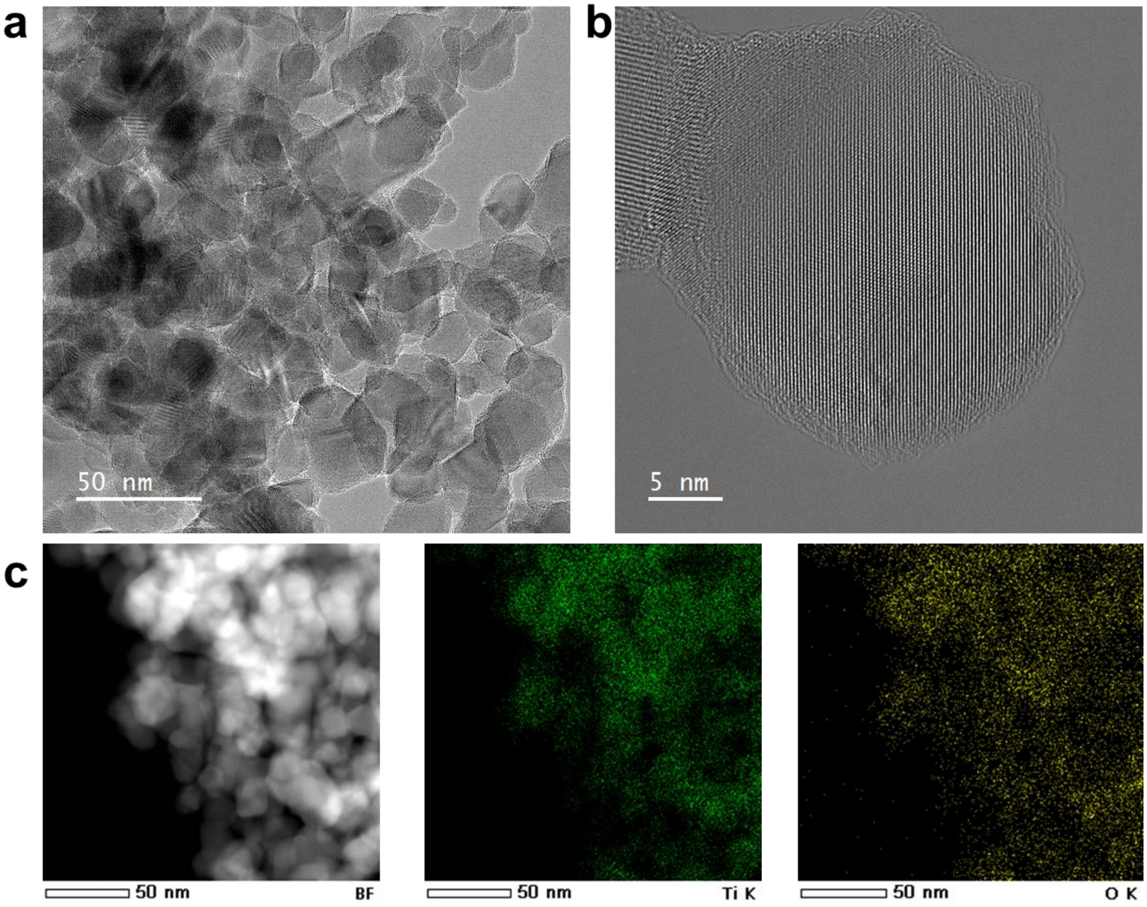

Supplementary Figure 1. TEM images of BTO. a TEM, **b** HR-TEM, and **c** HAADF-STEM images of BTO and EDS mappings of Ti and O.

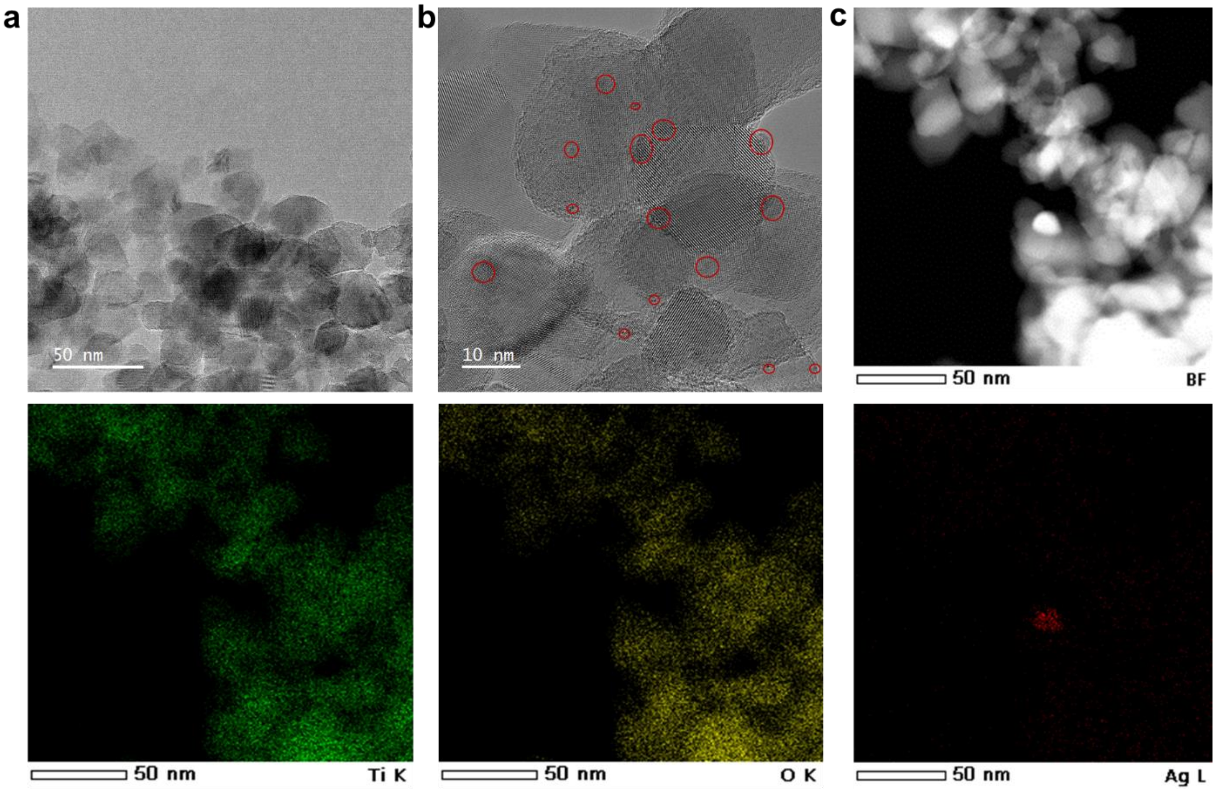

Supplementary Figure 2. TEM images of Ag-BTO. a TEM, **b** HR-TEM, and **c** HAADF-STEM images of Ag-BTO and EDS mappings of Ti, O, and Ag.

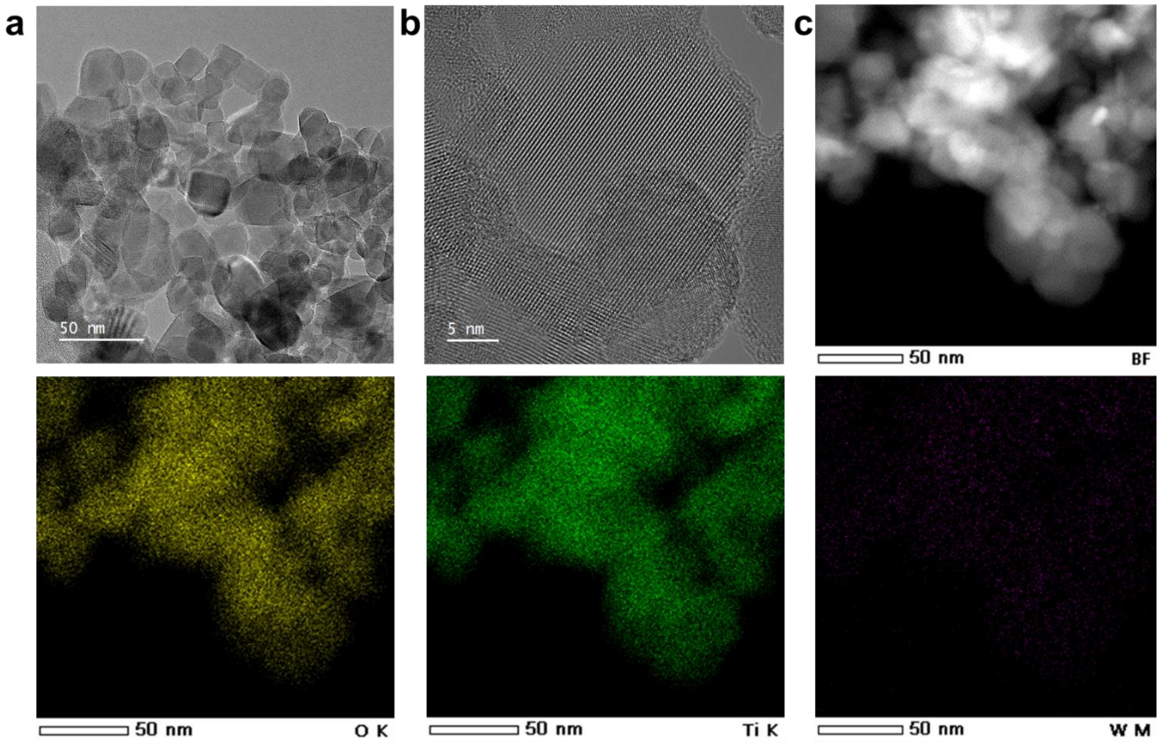

Supplementary Figure 3. TEM images of W-BTO. a TEM, **b** HR-TEM, and **c** HAADF-STEM images of W-BTO and EDS mappings of Ti, O, and W.

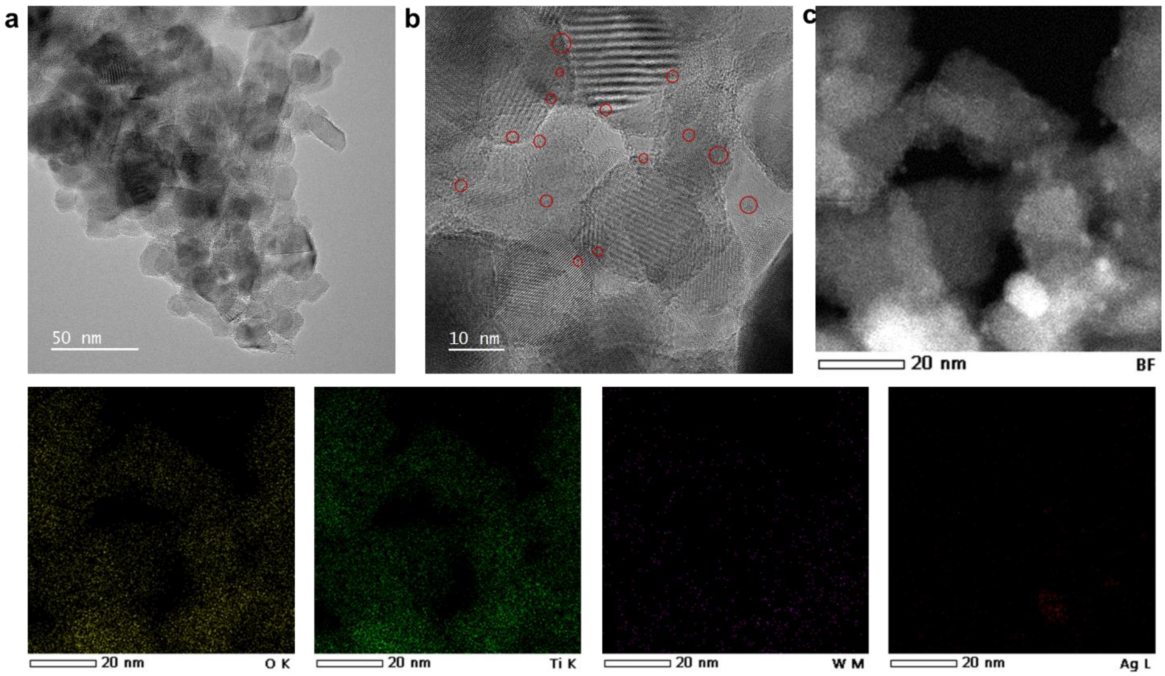

Supplementary Figure 4. TEM images of Ag.W-BTO-50. a TEM, **b** HR-TEM, and **c** HAADF-STEM images of Ag.W-BTO-50 and EDS mappings of Ti, O, W, and Ag.

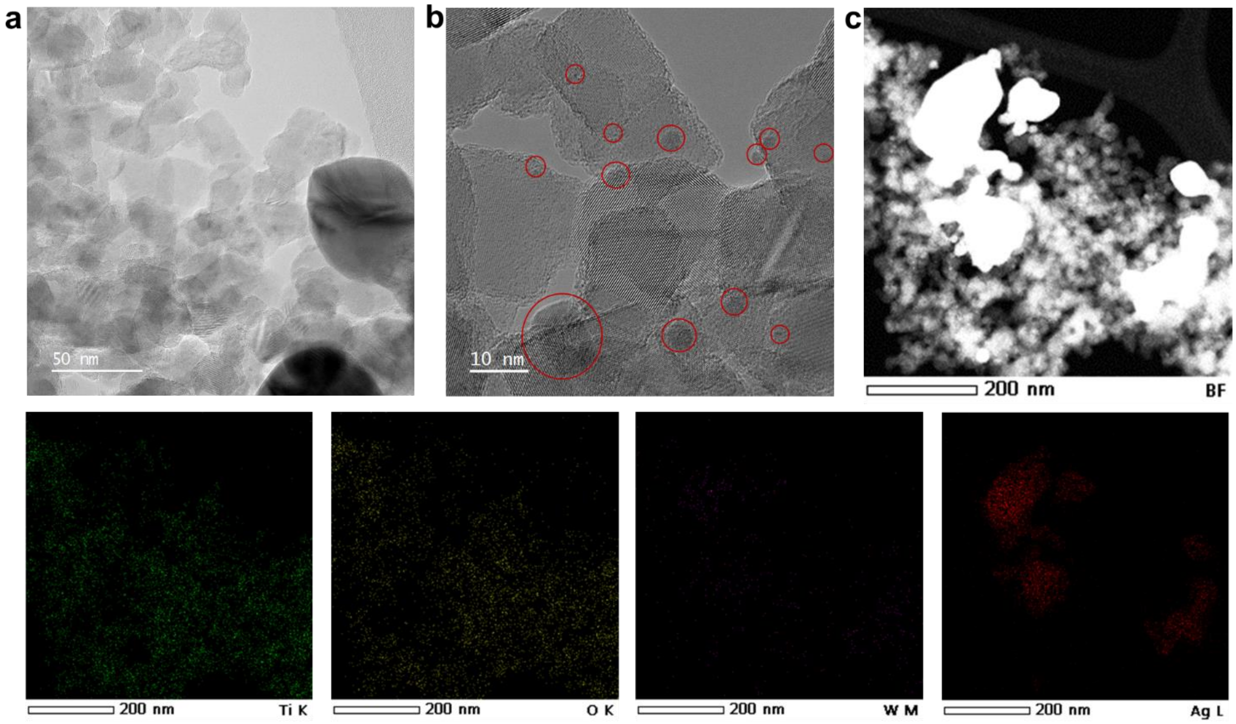

Supplementary Figure 5. TEM images of Ag.W-BTO-150. a TEM, **b** HR-TEM, and **c** HAADF-STEM images of Ag.W-BTO-150 and EDS mappings of Ti, O, W, and Ag.

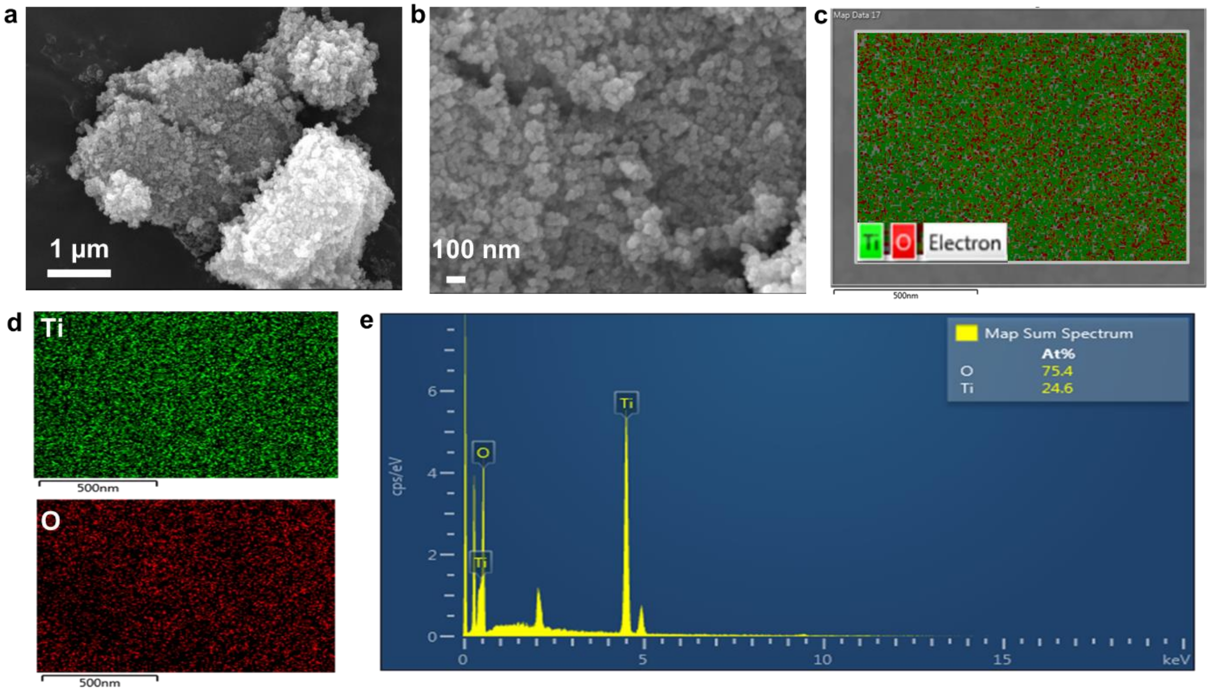

Supplementary Figure 6. SEM images of BTO. a, b SEM images of BTO nano-bulks. **c-e** Corresponding elemental mapping of Ti, O, and EDS of the selected area.

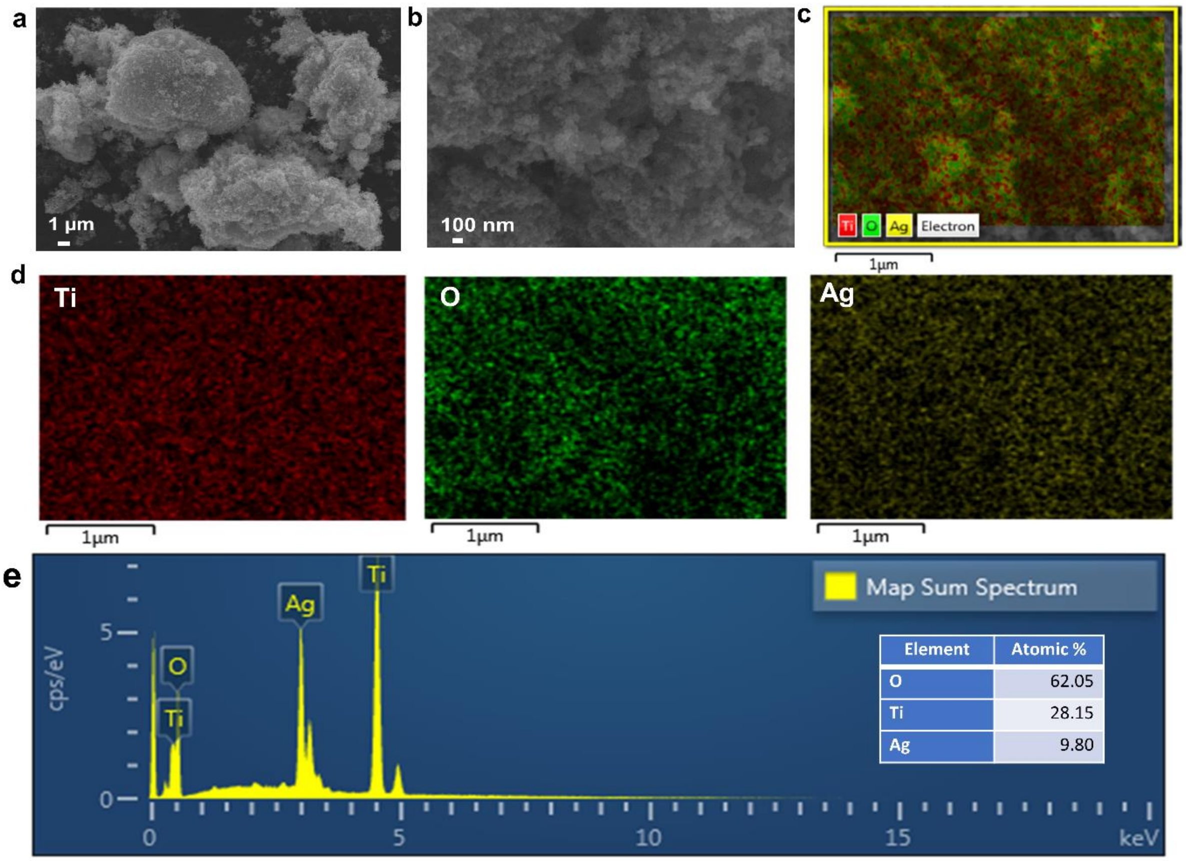

Supplementary Figure 7. SEM images of Ag-BTO. a, b SEM images of Ag-BTO nano-bulks. **c-e** Corresponding elemental mapping of Ag, Ti, O, and EDS of the selected area.

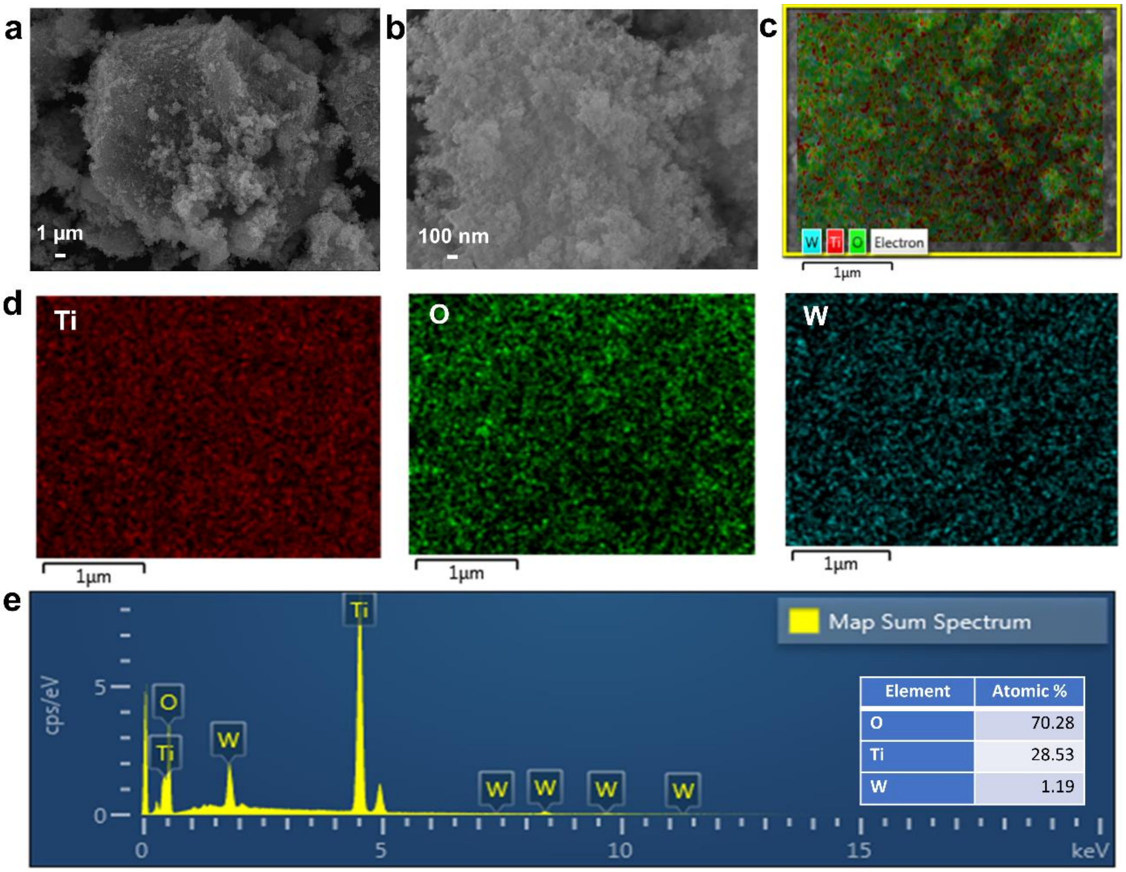

Supplementary Figure 8. SEM images of W-BTO. a, b SEM images of W-BTO nano-bulks. **c-e** Corresponding elemental mapping of W, Ti, O, and EDS of the selected area.

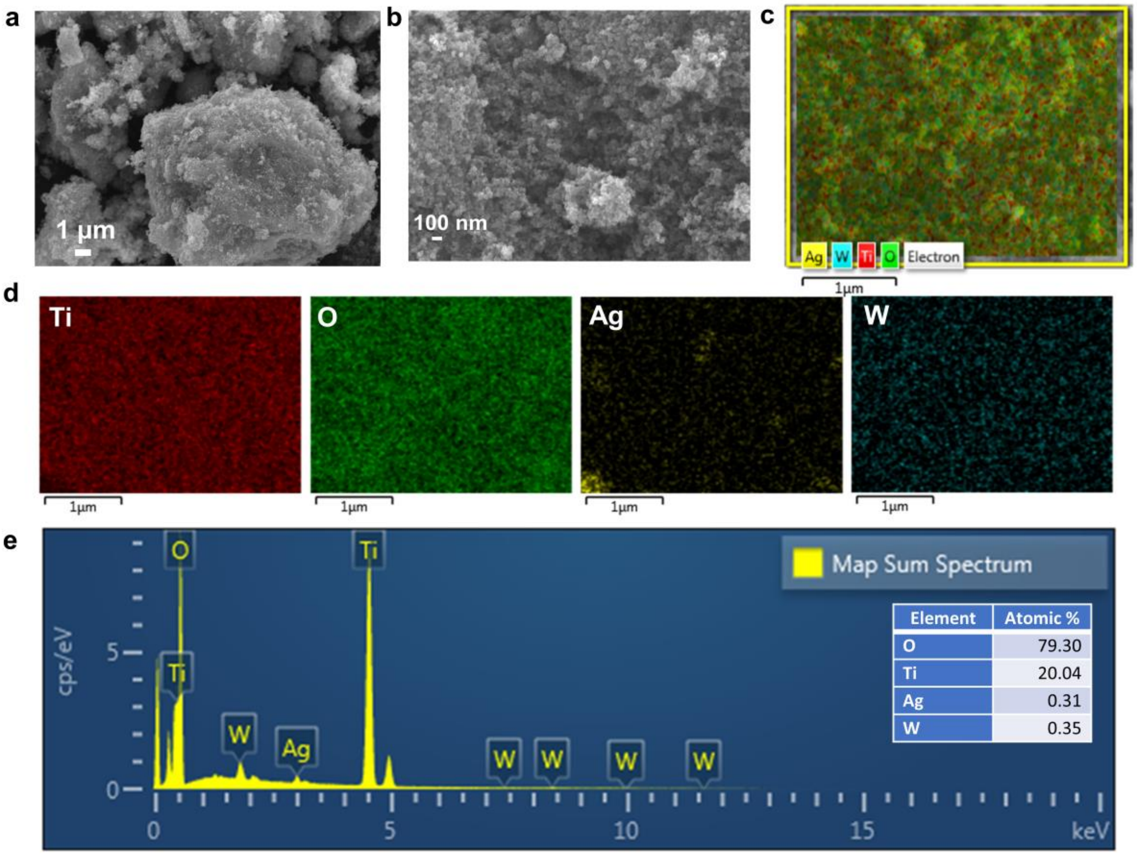

Supplementary Figure 9. SEM images of Ag.W-BTO-50. a, b SEM images of Ag.W-BTO-50 nano-bulks. **c-e** Corresponding elemental mapping of Ag, W, Ti, O, and EDS of the selected area.

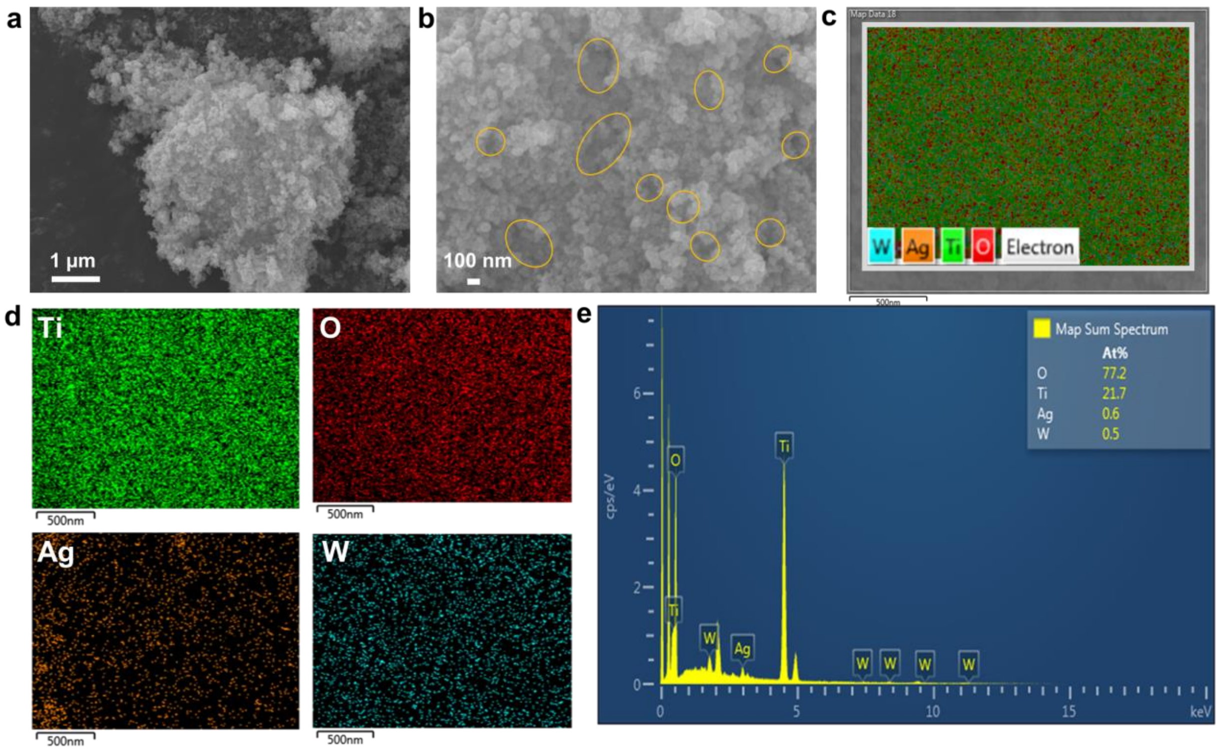

Supplementary Figure 10. SEM images of Ag.W-BTO. a, b SEM images of Ag.W-BTO nano-bulks. **c-e** Corresponding elemental mapping of Ti, O, Ag, W, and EDS of the selected area.

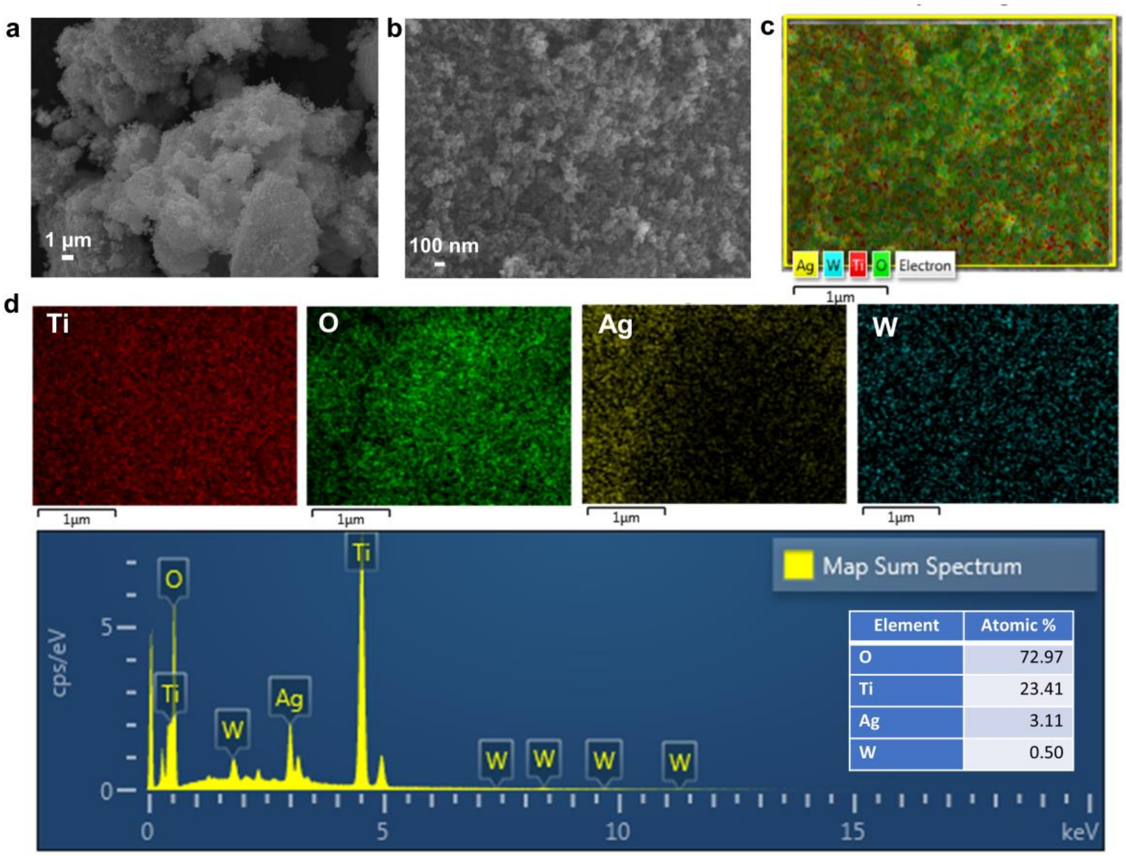

Supplementary Figure 11. SEM images of Ag.W-BTO-150. a, b SEM images of Ag.W-BTO-150 nano-bulks. **c-e** Corresponding elemental mapping of Ti, O, Ag, W, and EDS of the selected area.

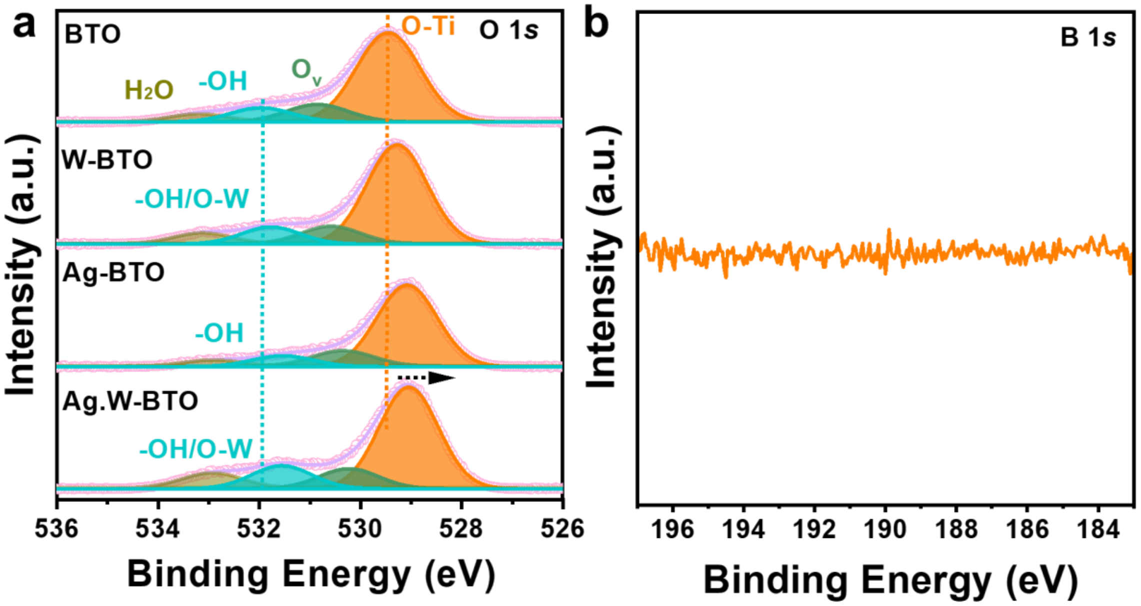

Supplementary Figure 12. XPS spectra. a O 1*s* XPS of BTO, W-BTO, Ag-BTO, and Ag.W-BTO, respectively. **b** B 1*s* XPS of Ag.W-BTO.

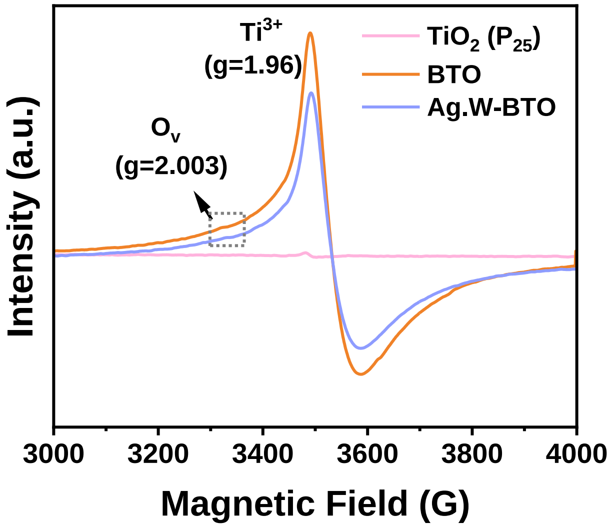

Supplementary Figure 13. Electron paramagnetic resonance (EPR) spectra of TiO_2 (P_{25}), BTO and Ag.W-BTO.

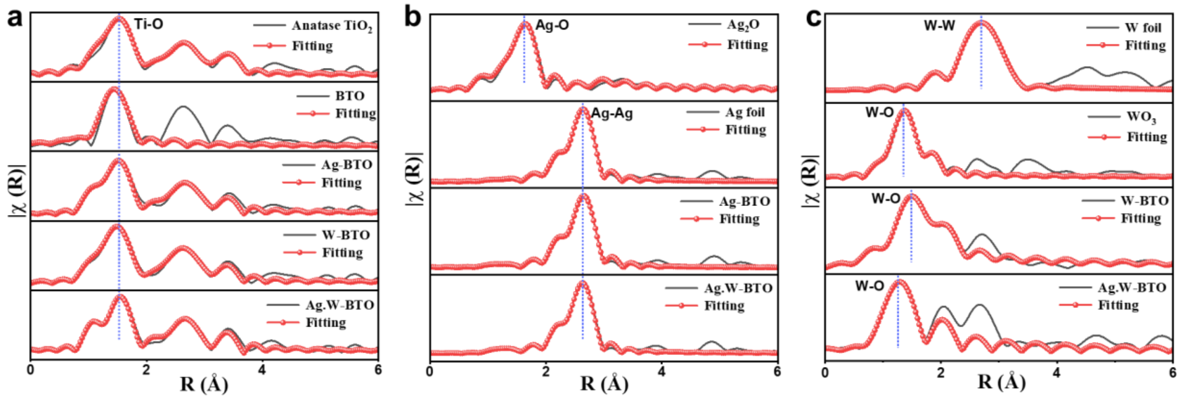

Supplementary Figure 14. Fitting EXAFS spectrum. a-c Extended X-ray absorption fine structure (EXAFS) k^3 $\chi(k)$ Fourier-transform (FT) and fitting spectra of Ti, Ag, and W in R-space of catalysts with standard references, respectively.

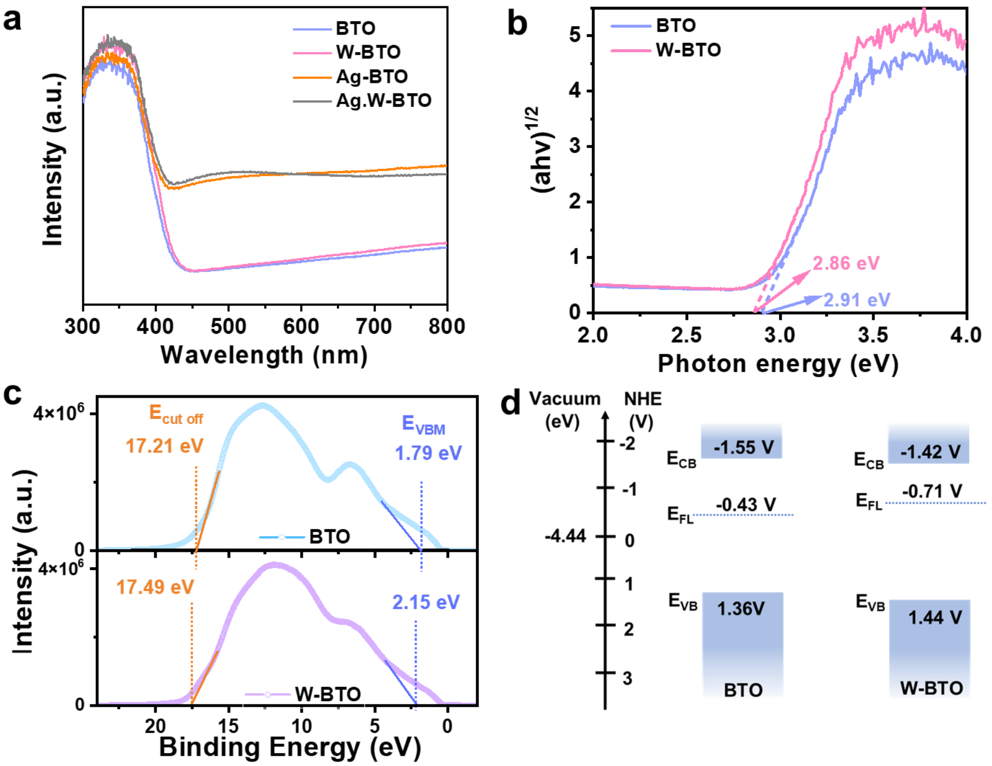

Supplementary Figure 15. Band gap structures. **a** UV-vis of BTO, W-BTO, Ag-BTO, and Ag.W-BTO. **b** estimation of band gap, **c** UPS and **d** band gap structure of BTO, W-BTO.

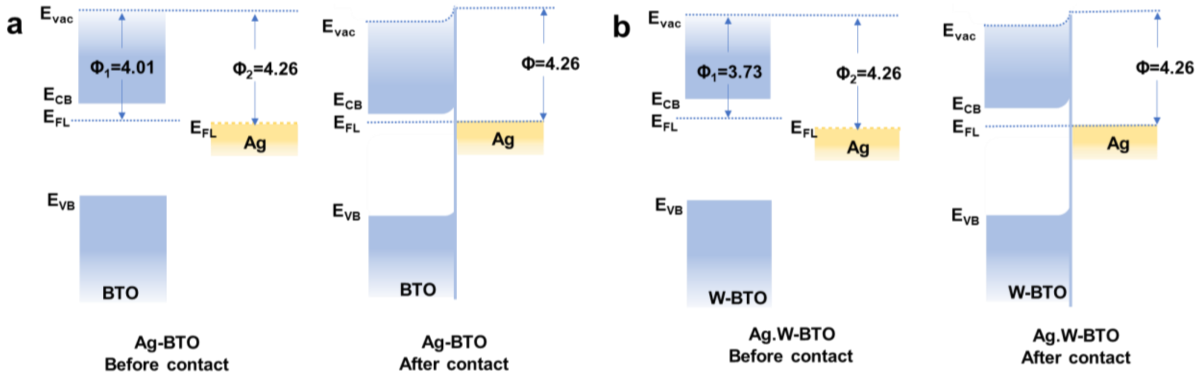

Supplementary Figure 16. Band gap structures. a Ag-BTO and **b** Ag.W-BTO.

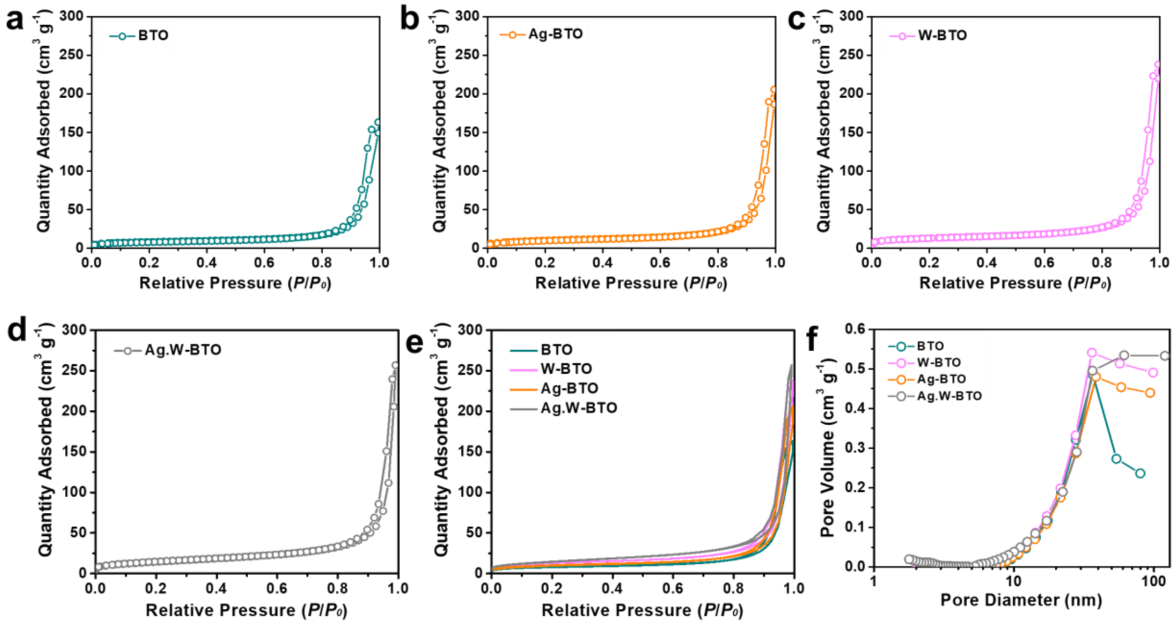

Supplementary Figure 17. N_2 sorption isotherms. a BTO, **b** Ag-BTO, **c** W-BTO and **d** Ag.W-BTO. **e** Comparison N_2 sorption isotherms and **f** pore sizes of BTO, W-BTO, Ag-BTO, and Ag.W-BTO.

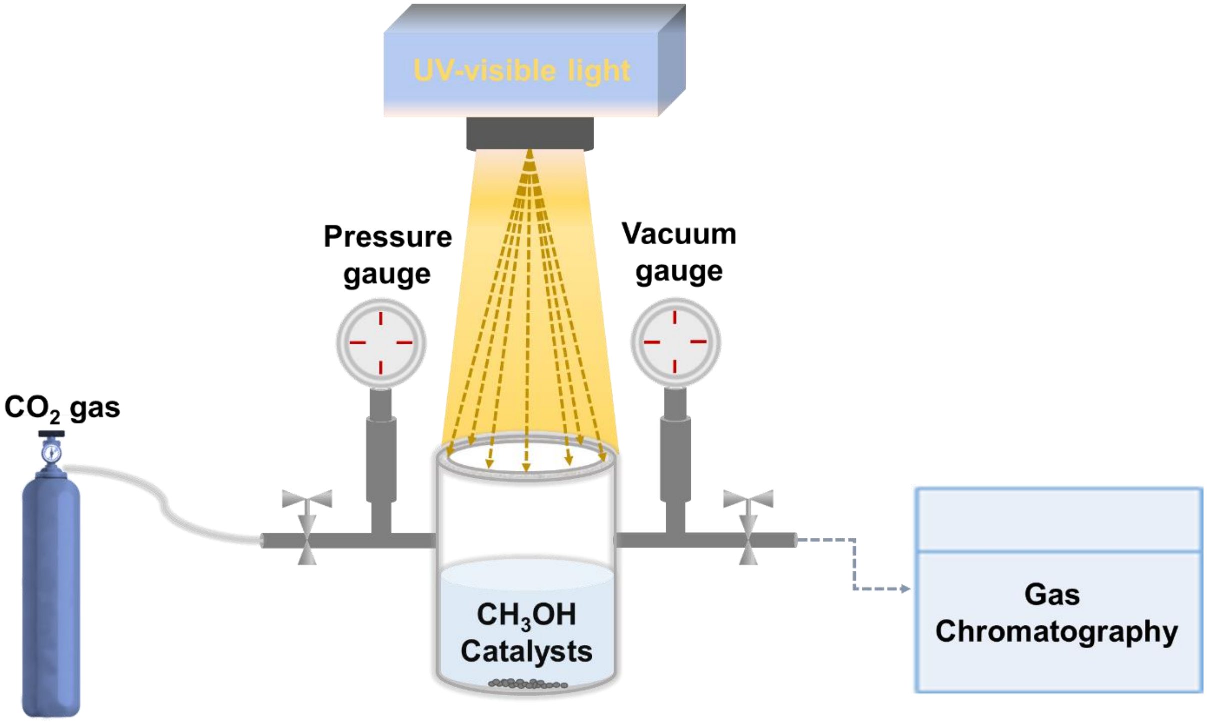

Supplementary Figure 18. Experimental setup used for photocatalytic reaction.

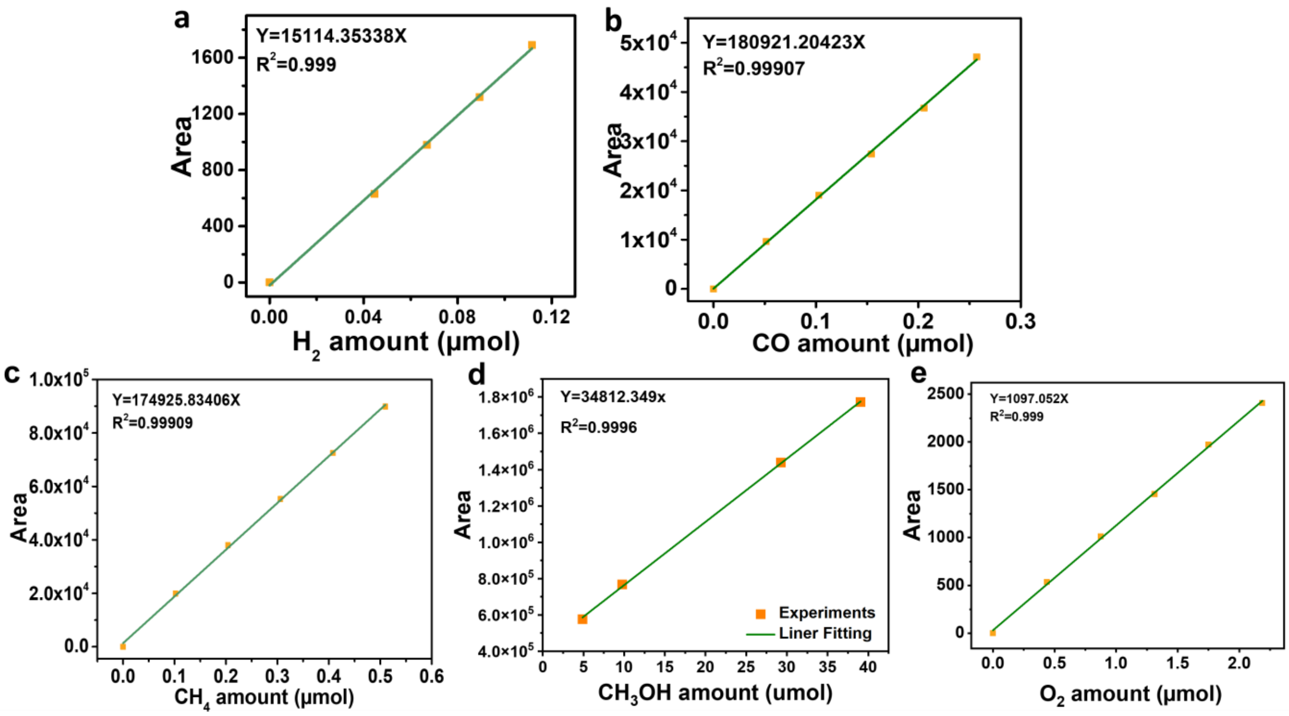

Supplementary Figure 19. Calibration curves of GC for the quantification of standard. **a** H_2, **b** CO, **c** CH_4, **d** CH_3OH, and **e** O_2.

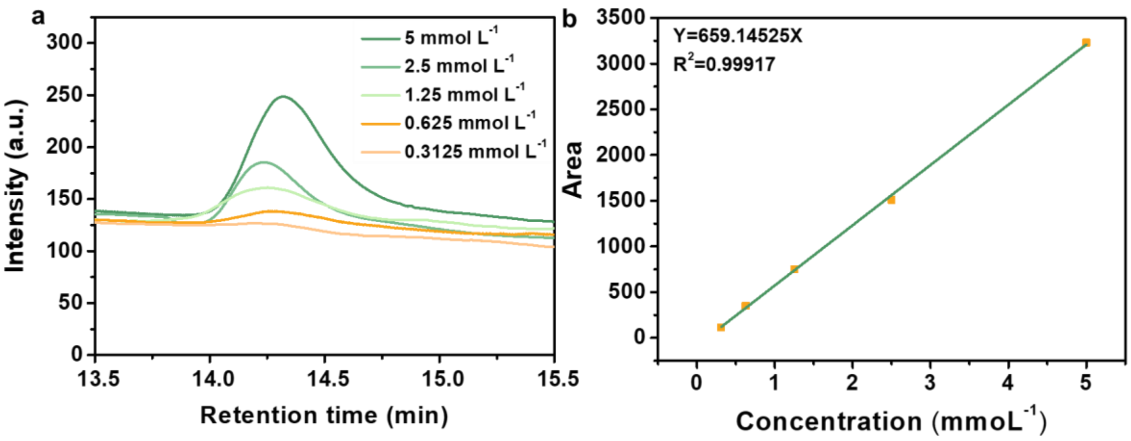

Supplementary Figure 20. Calibration curves of CH_2O. **a** GC spectra of the different amounts of CH_2O standard samples and **b** calibration curves of GC for the quantification of CH_2O.

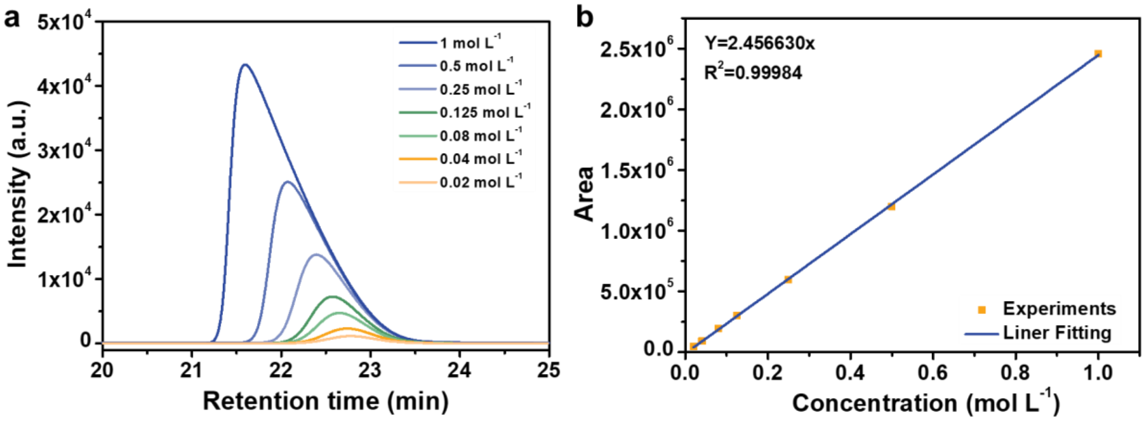

Supplementary Figure 21. Calibration curves of DMM. a GC spectrum of DMM standard samples and **b** calibration curves of GC for the quantification of DMM.

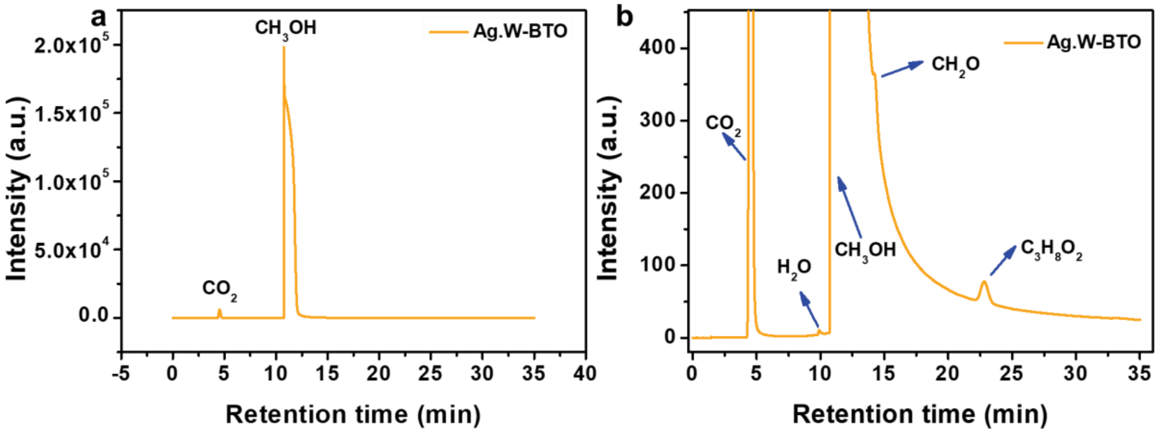

Supplementary Figure 22. Results of GC spectra. a Total GC spectra and **b** Enlargement GC spectra of Ag.W-BTO from 0 to 35 min of retention time. Enlargement of GC spectra more clearly confirms the products after CO_2 reduction on Ag.W-BTO, which shows ultra-high selectivity of DMM.

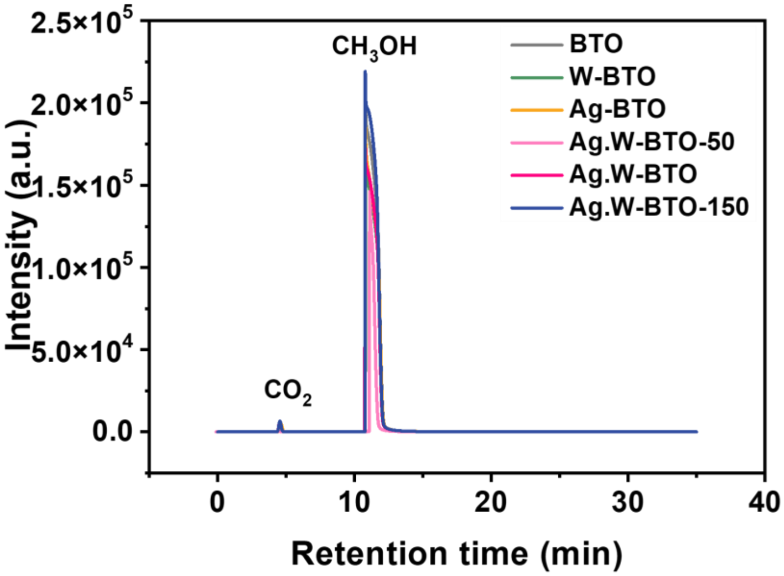

Supplementary Figure 23. Results of GC spectra. Total GC spectrum of all prepared samples from 0 to 35 min of retention time. Due to the large amount of CH_3OH, it is difficult to find the peak of production in the same spectra, so the enlarged spectra of production at a specific retention time after CO_2 reduction are shown in Supplementary Figure 24.

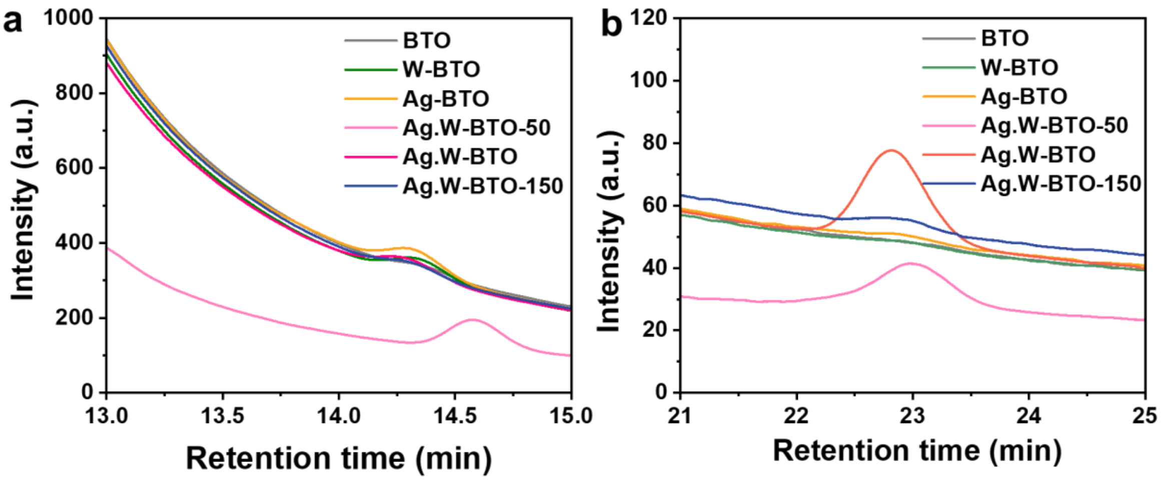

Supplementary Figure 24. Results of GC spectra. a GC spectrum (CH_2O) of all prepared samples from 13 to 15 min of retention time. **b** GC spectra (DMM) of all prepared samples from 21 to 25 min of retention time.

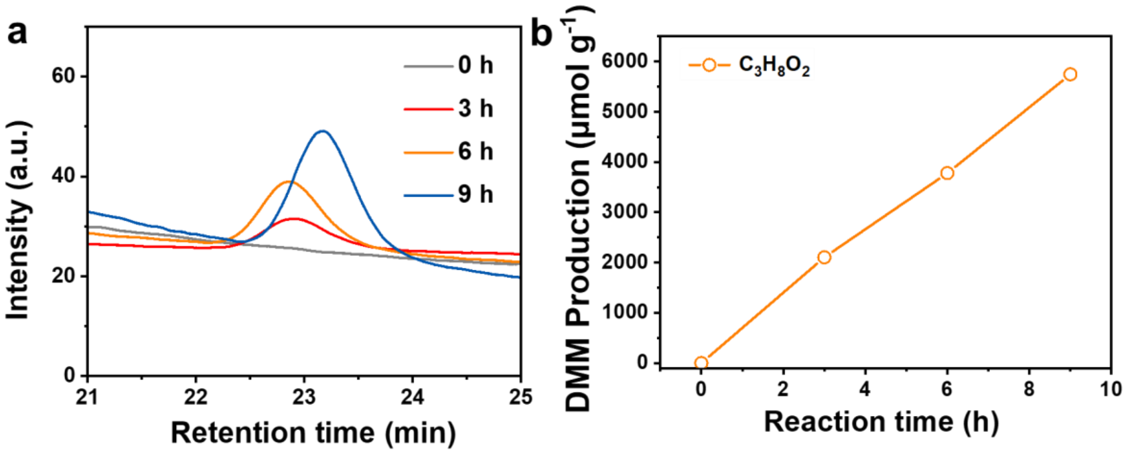

Supplementary Figure 25. Results of GC spectra. a GC spectra (DMM) on Ag.W-BTO from 21 to 25 min of retention time. **b** the in-operando performance of DMM in 9 h.

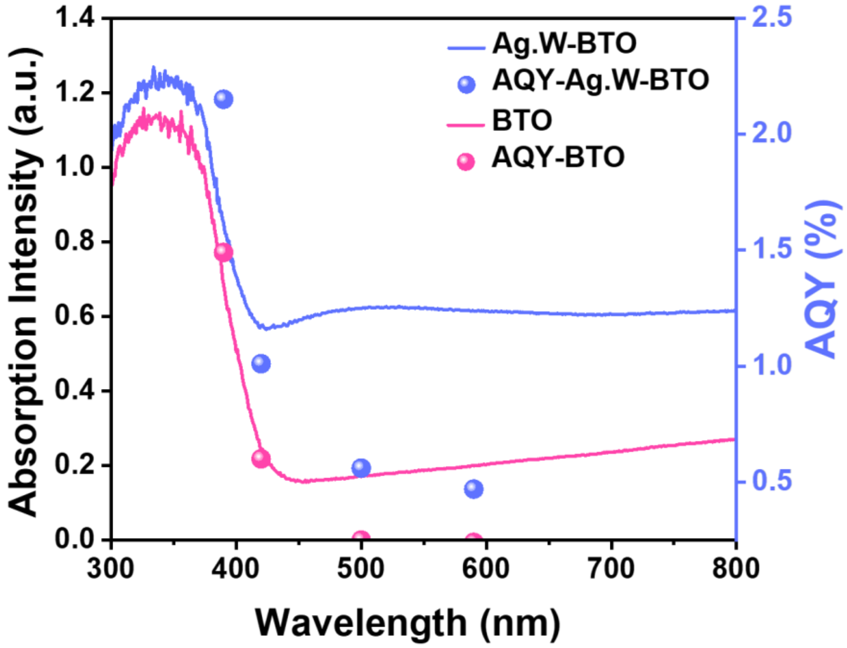

Supplementary Figure 26. UV-vis absorption spectrum and wavelength-dependent AQY of BTO and Ag.W-BTO.

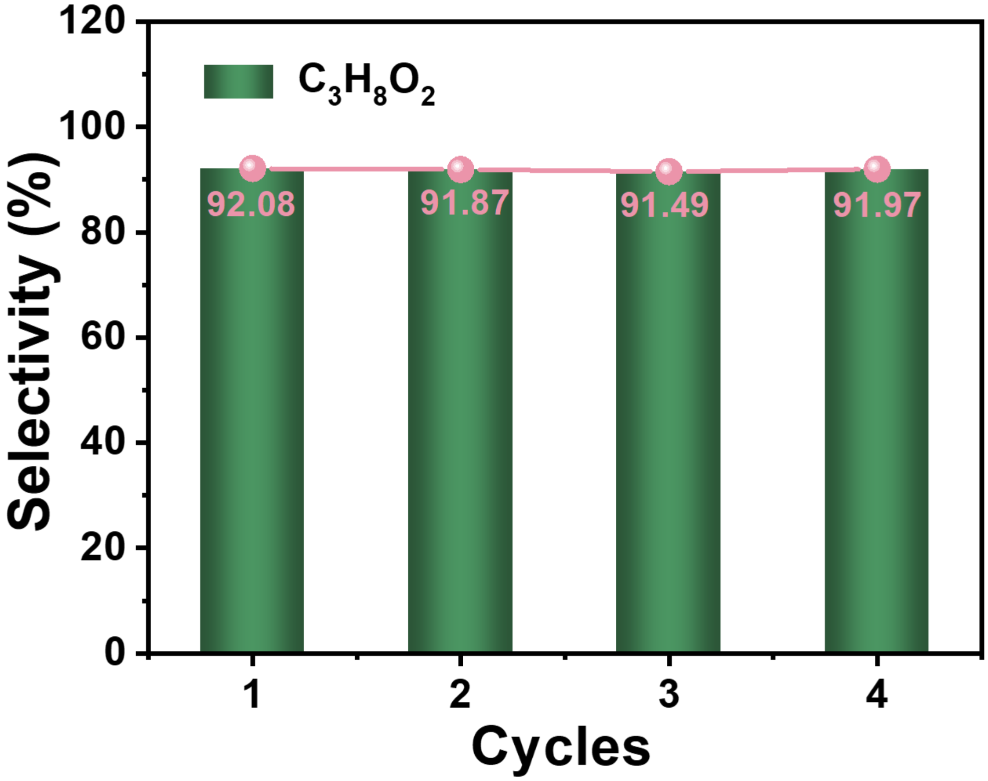

Supplementary Figure 27. The cycling measurements of DMM stability on Ag.W-BTO.

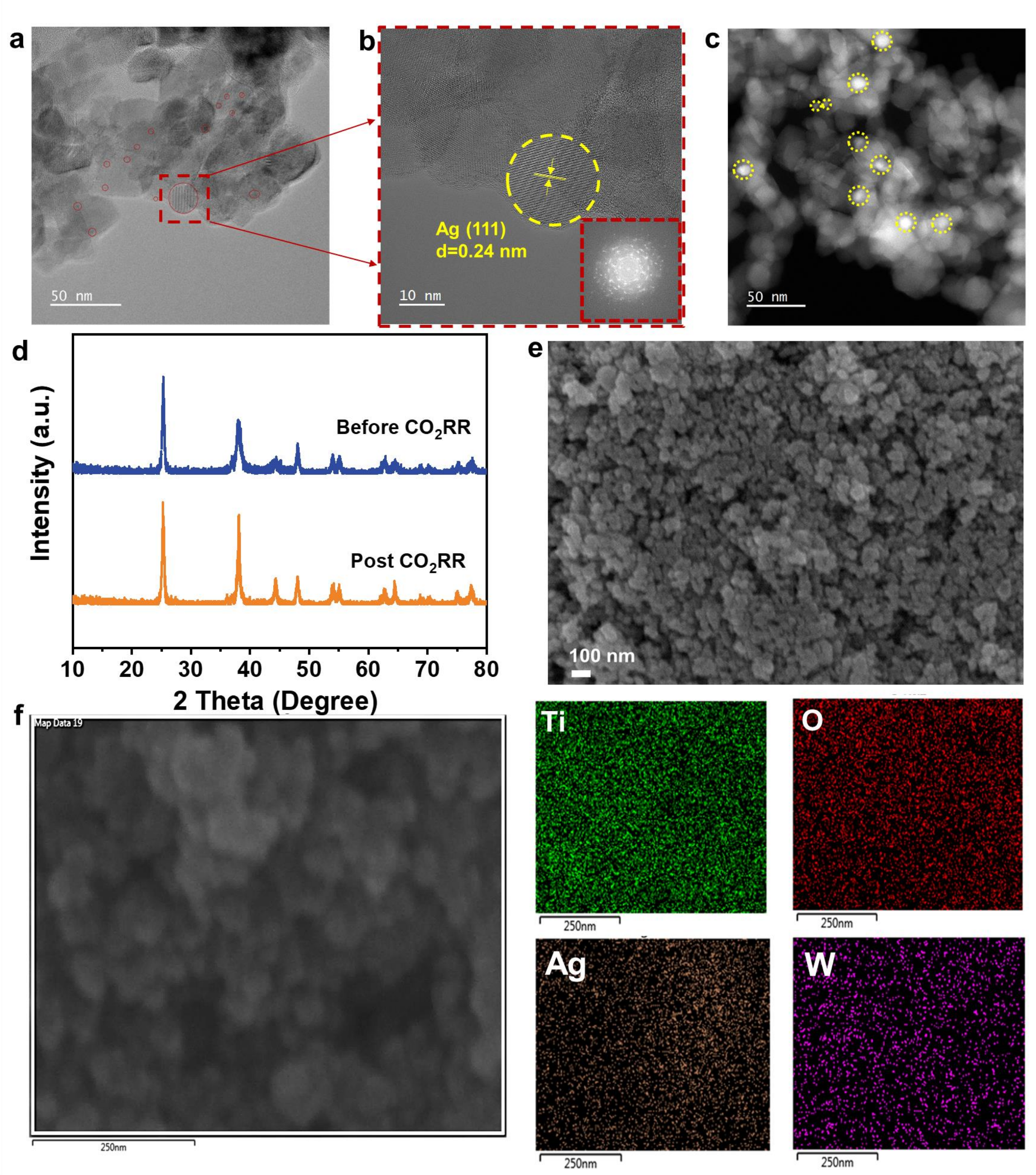

Supplementary Figure 28. Morphology and structural characterizations of Ag.W-BTO after photocatalytic stability test. a-c TEM, HR-TEM with FFT pattern and STEM images of Ag.W-BTO after stability test. **d** XRD patterns of before and post CO_2RR reaction on Ag.W-BTO. **e,f** SEM and SEM-EDS mapping Ti, O, Ag, W of Ag.W-BTO.

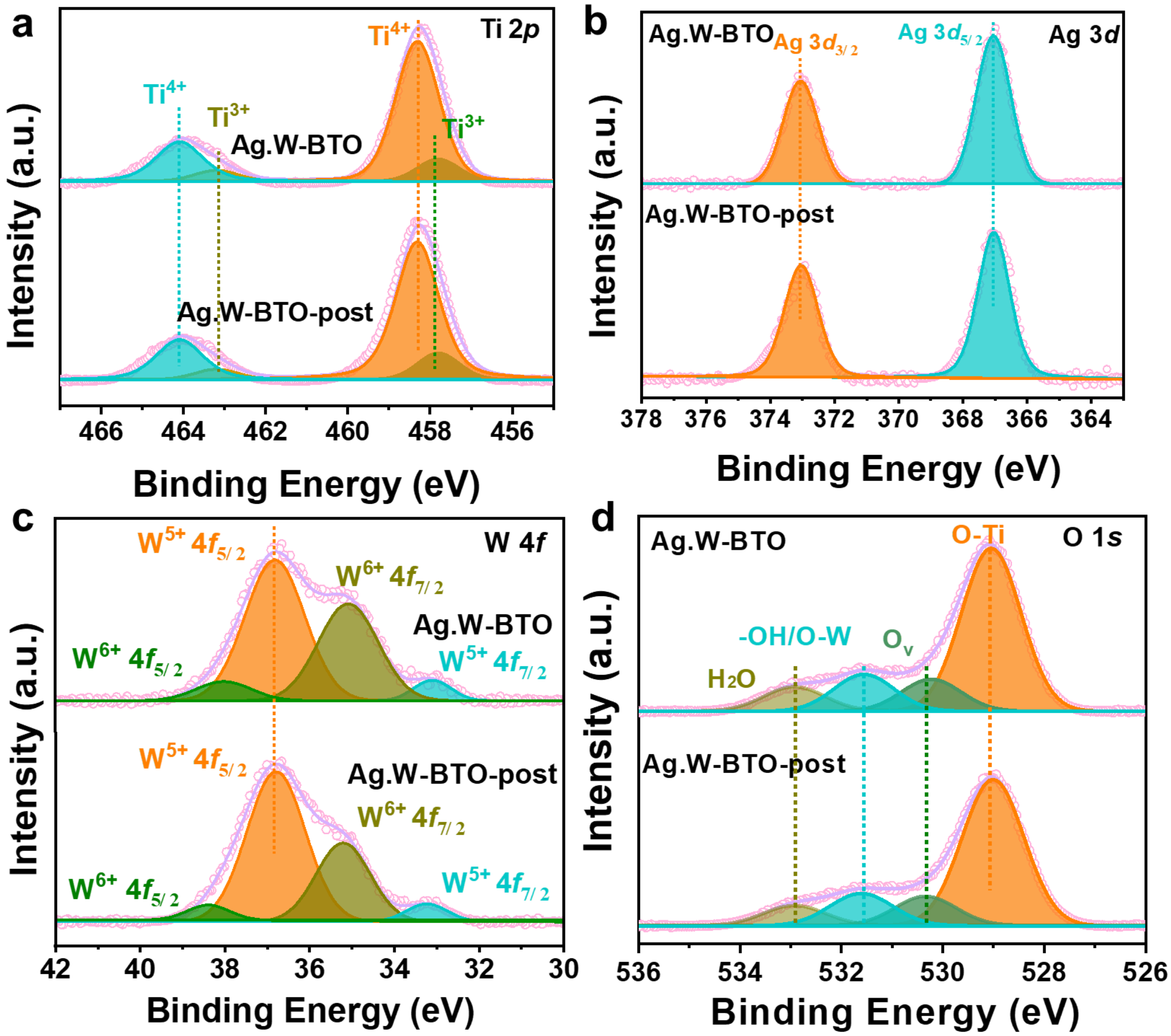

Supplementary Figure 29. XPS of Ag.W-BTO before and after the stability. a Ti 2*p*, **b** Ag 3*d*, **c** W 4*f*, and **d** O 1*s*.

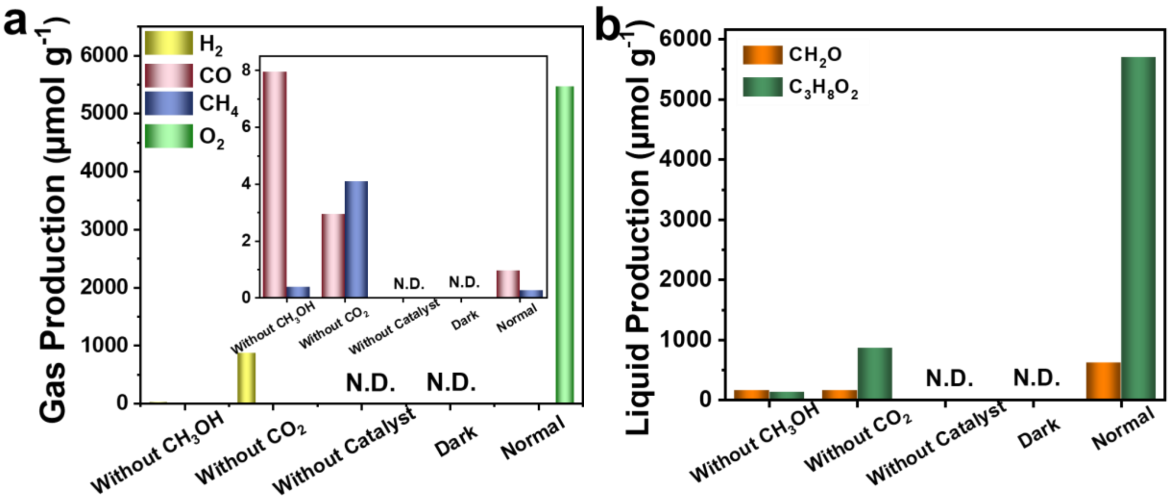

Supplementary Figure 30. Control experiments of CO_2RR in 9h on Ag.W-BTO catalyst under different reaction conditions. a gas production rate, **b** liquid production rate. (N.D. means no detection).

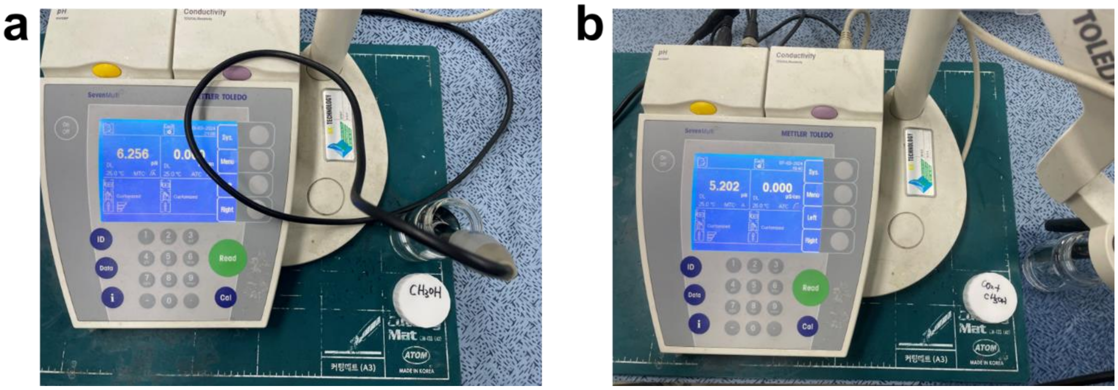

Supplementary Figure 31. The pH values comparison of CH_3OH by a pH meter. a before and **b** after flowing CO_2 gas.

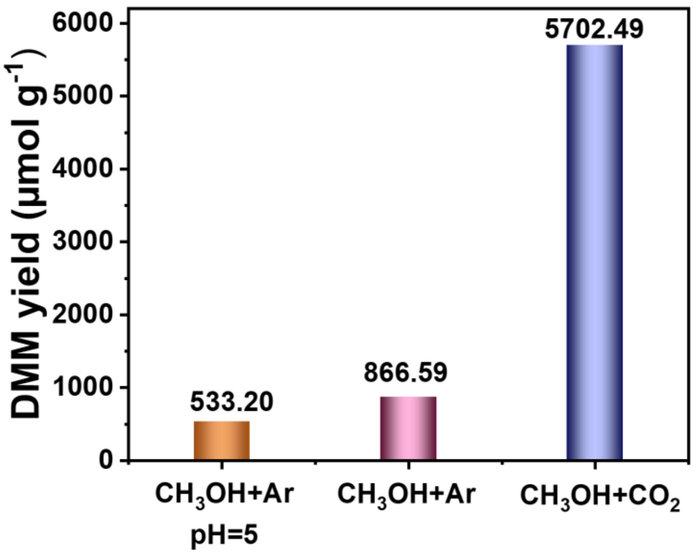

Supplementary Figure 32. The result of pH impact on DMM yield.

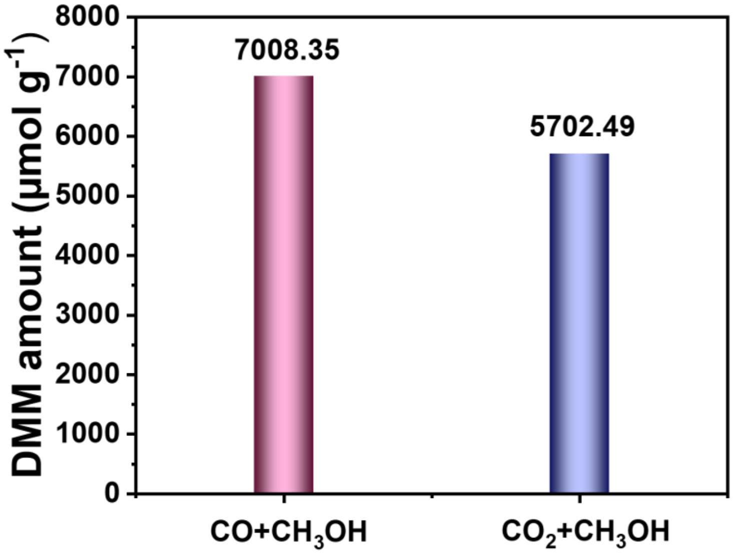

Supplementary Figure 33. The comparison of DMM production amount of CO + CH_3OH and CO_2 + CH_3OH.

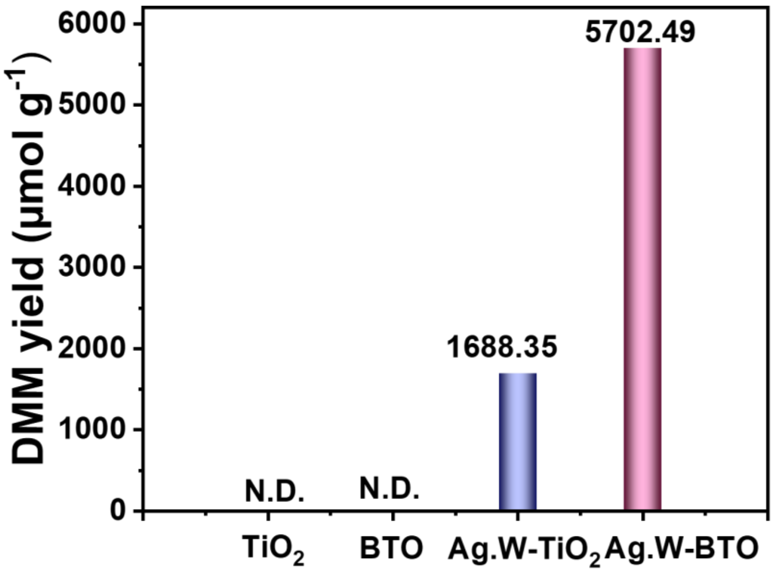

Supplementary Figure 34. The comparison of DMM production amount on TiO_2, BTO, Ag.W-TiO_2 and Ag.W-BTO, respectively.

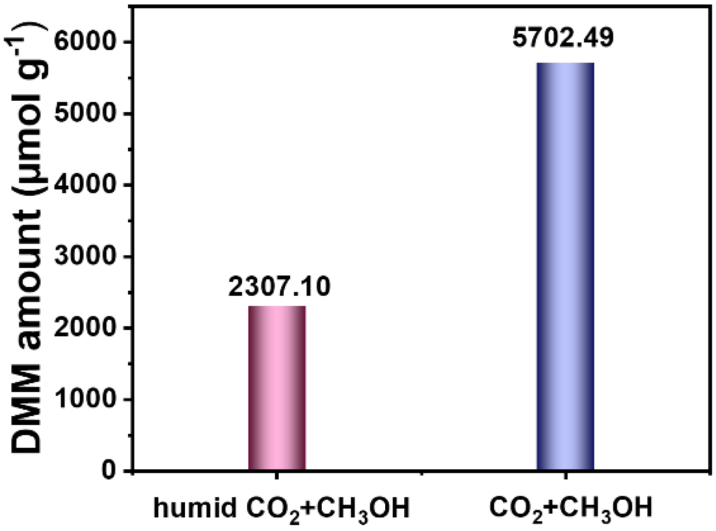

Supplementary Figure 35. The comparison of DMM production amount of humid CO_2 + CH_3OH and CO_2 + CH_3OH.

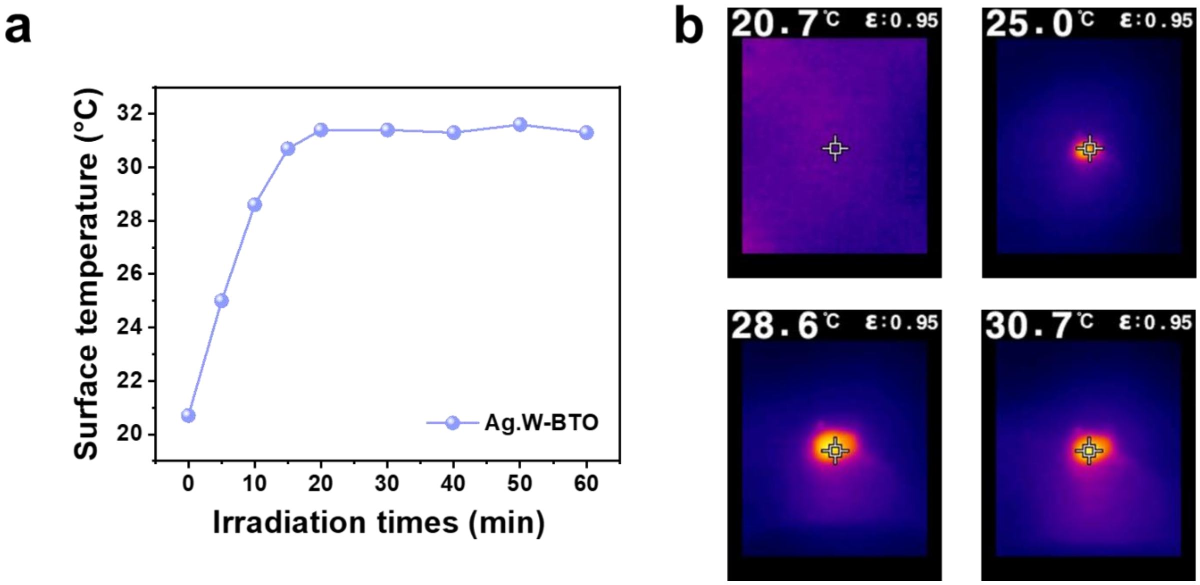

Supplementary Figure 36. Localized surface plasmon resonance (LSPR) effect of Ag.W-BTO. **a** Temperature vs time curves of Ag.W-BTO within 60 min. **b** Representative infrared thermal image.

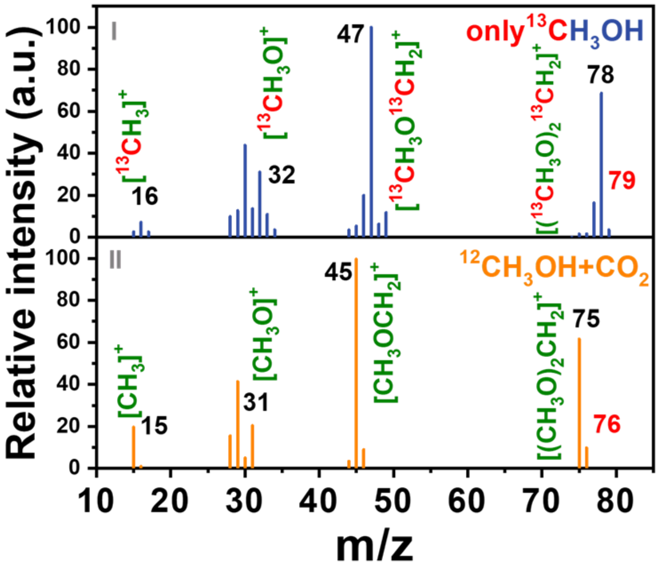

Supplementary Figure 37. GC-MS result of I) only isotope labeled $^{13}CH_3OH$ and II) isotope non-labeled $^{12}CH_3OH$ + CO_2.

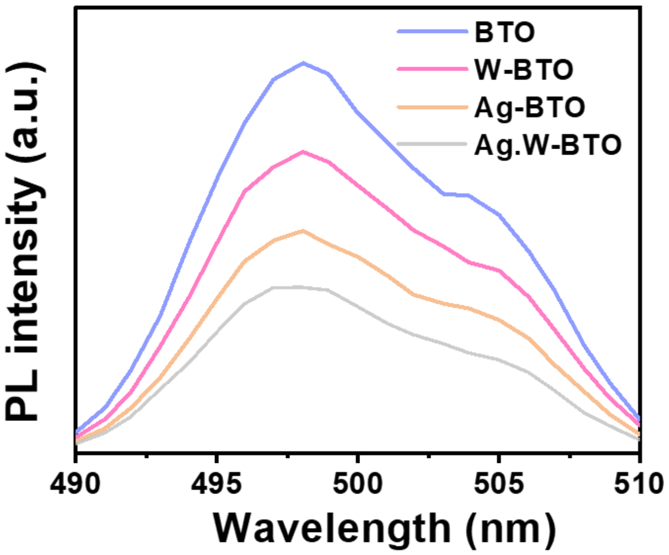

Supplementary Figure 38. PL of BTO, W-BTO, Ag-BTO, and Ag.W-BTO.

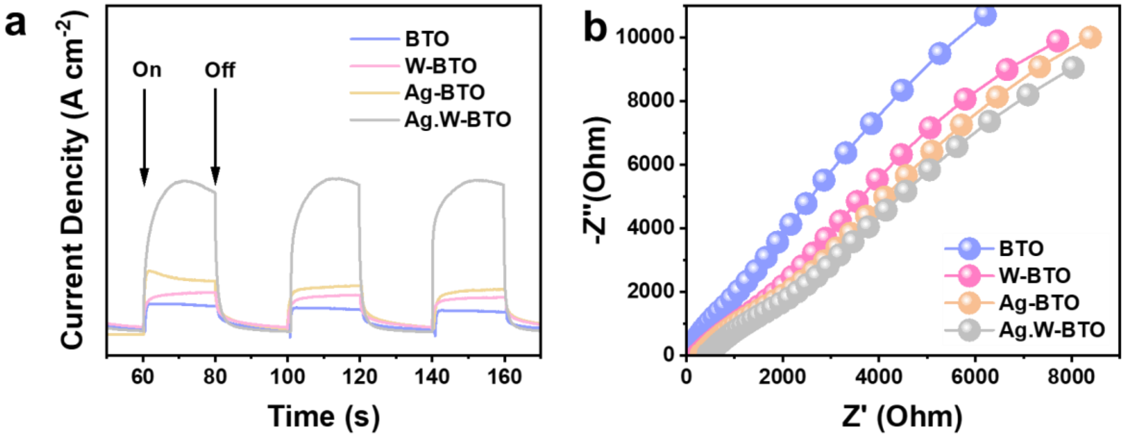

Supplementary Figure 39. Photoelectrochemical characterization. a Photo-current and **b** Electrochemical impedance spectroscopy (EIS) of BTO, W-BTO, Ag-BTO, and Ag.W-BTO.

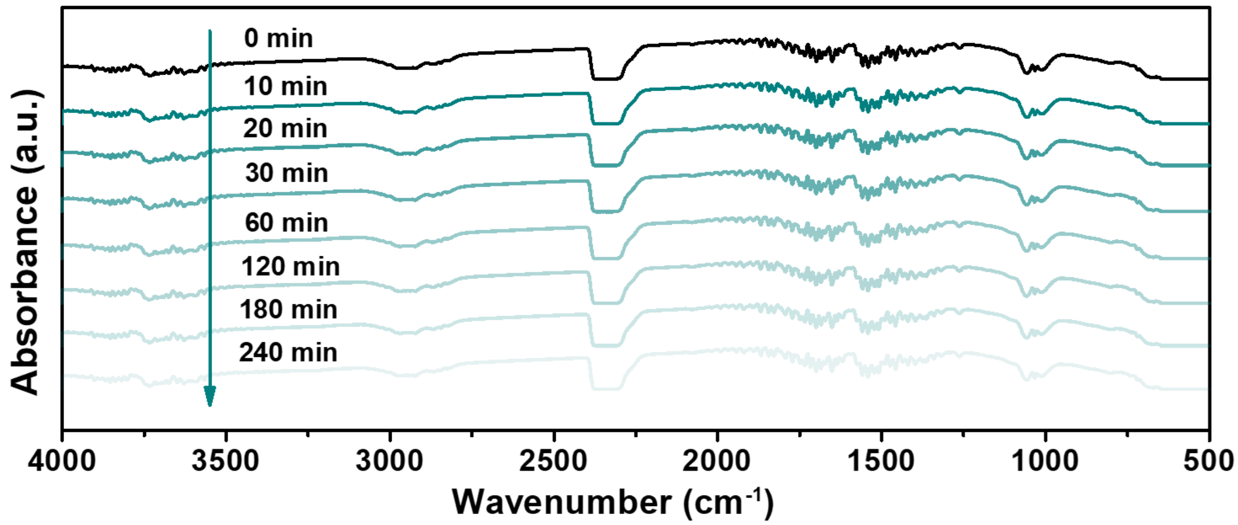

Supplementary Figure 40. In-situ DRIFTS spectra of KBr background.

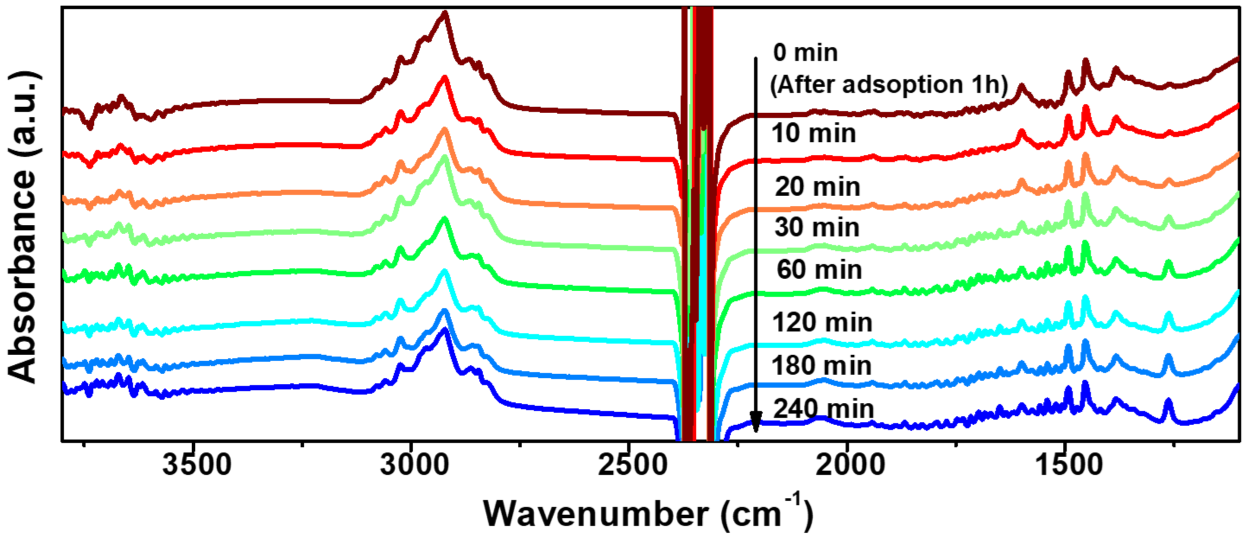

Supplementary Figure 41. In-situ DRIFTS spectra of CO_2RR with MOR on Ag.W-BTO.

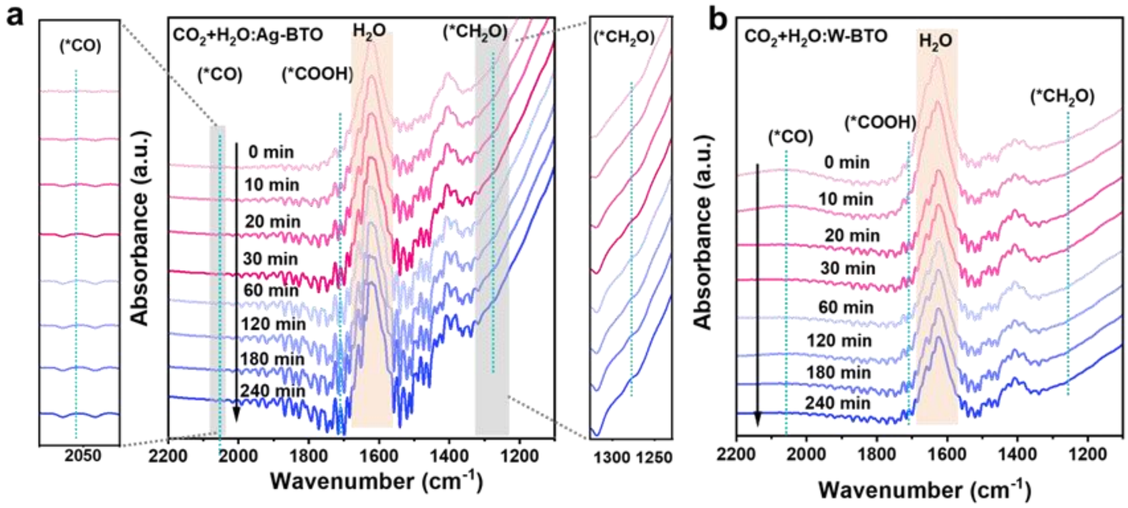

Supplementary Figure 42. The comparison of in-situ DRIFTS spectra by flowing CO_2 + H_2O. a Ag-BTO with enlarged view of shaded area. **b** W-BTO under UV-visible light irradiation in different reaction times.

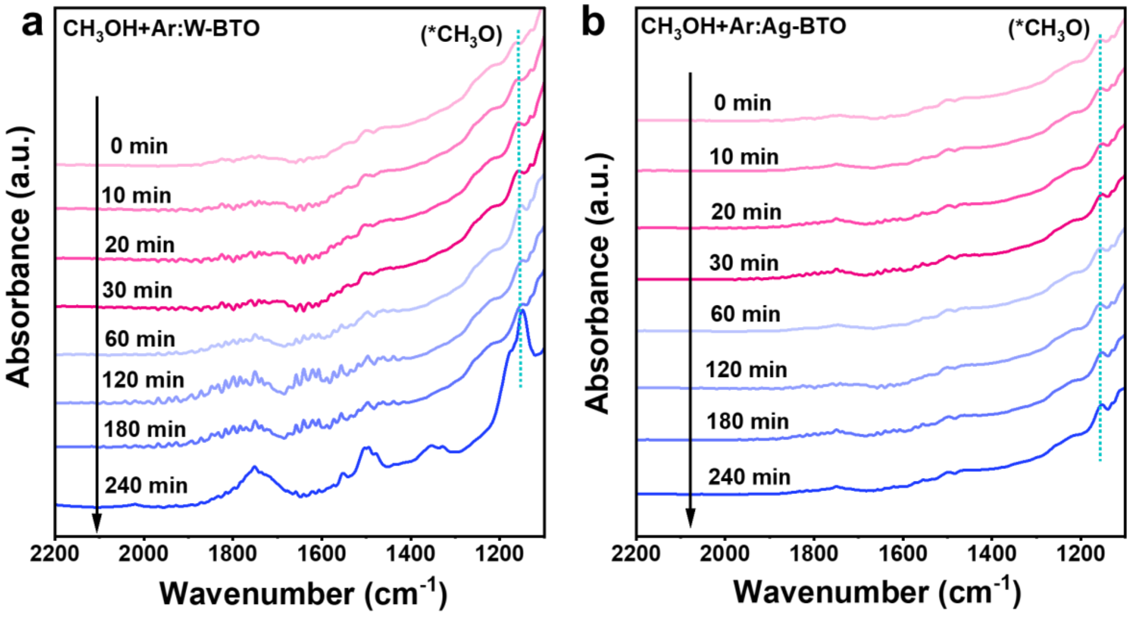

Supplementary Figure 43. The comparison of in-situ DRIFTS spectra by flowing CH_3OH + Ar. a W-BTO and **b** Ag-BTO under UV-visible light irradiation in different reaction times.

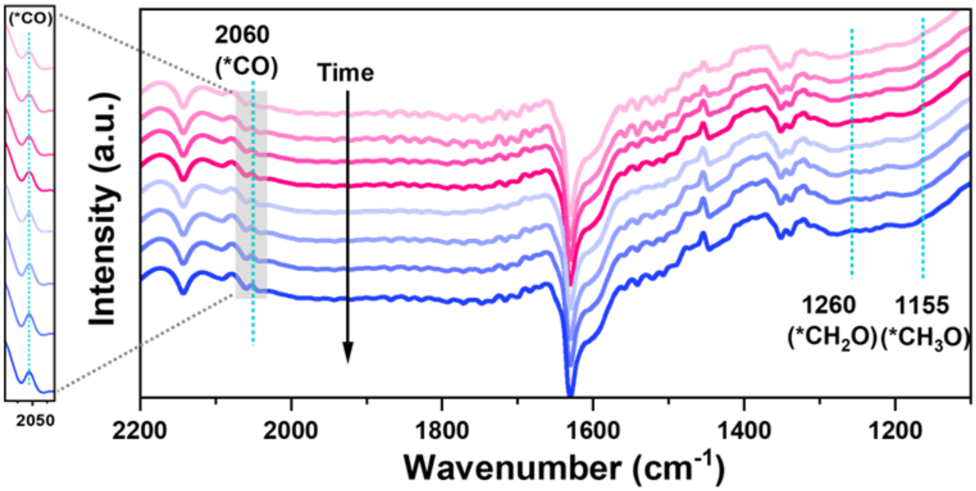

Supplementary Figure 44. In-situ DRIFTS spectra on the Ag.W-BTO by flowing CO and CH_3OH gas under UV-visible irradiation at detailed reaction times (0, 10, 20, 30, 60, 120, 180, 240 min) after adoption 1 hour in the dark.

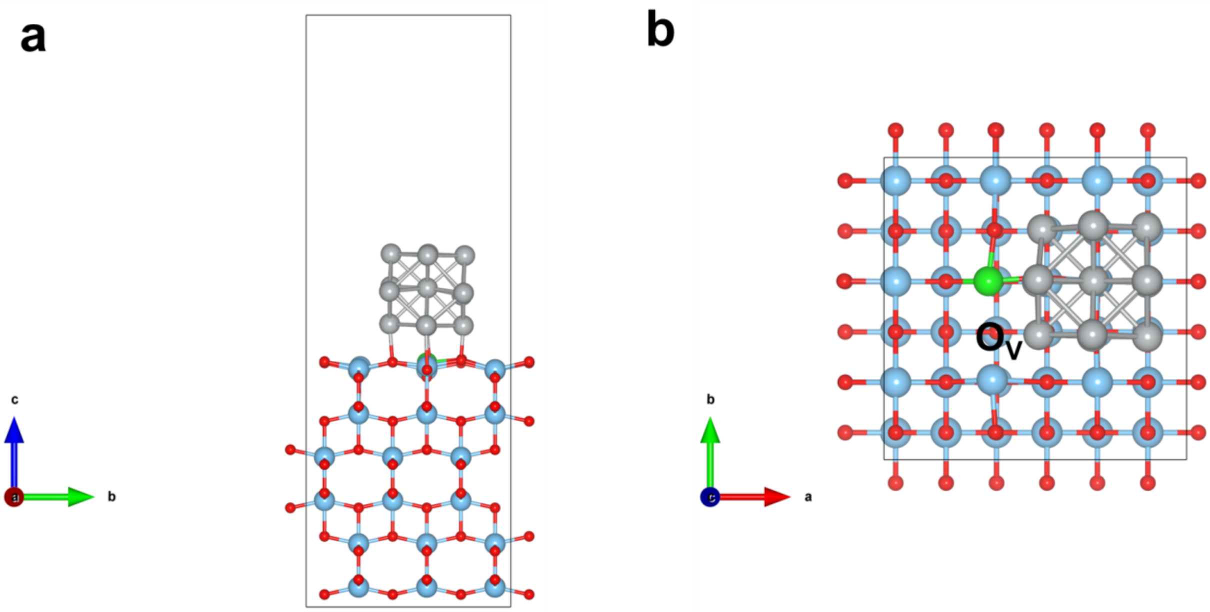

Supplementary Figure 45. The optimized Ag.W-BTO structure. a side view and **b** top view.

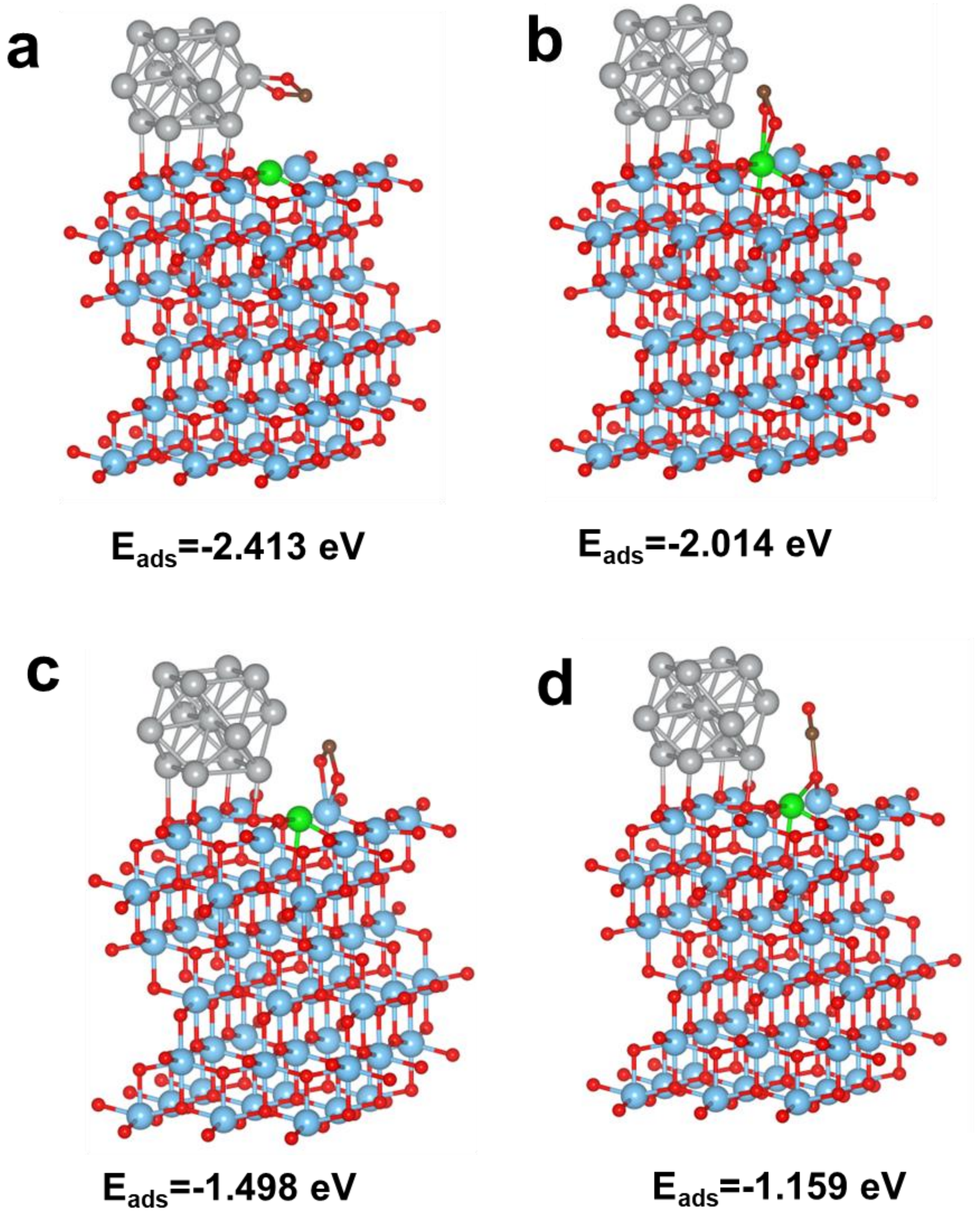

Supplementary Figure 46. Optimized DFT structures for CO_2 adsorption. a Ag, **b** W, **c** Ti, and **d** O_v active sites of Ag.W-BTO, respectively.

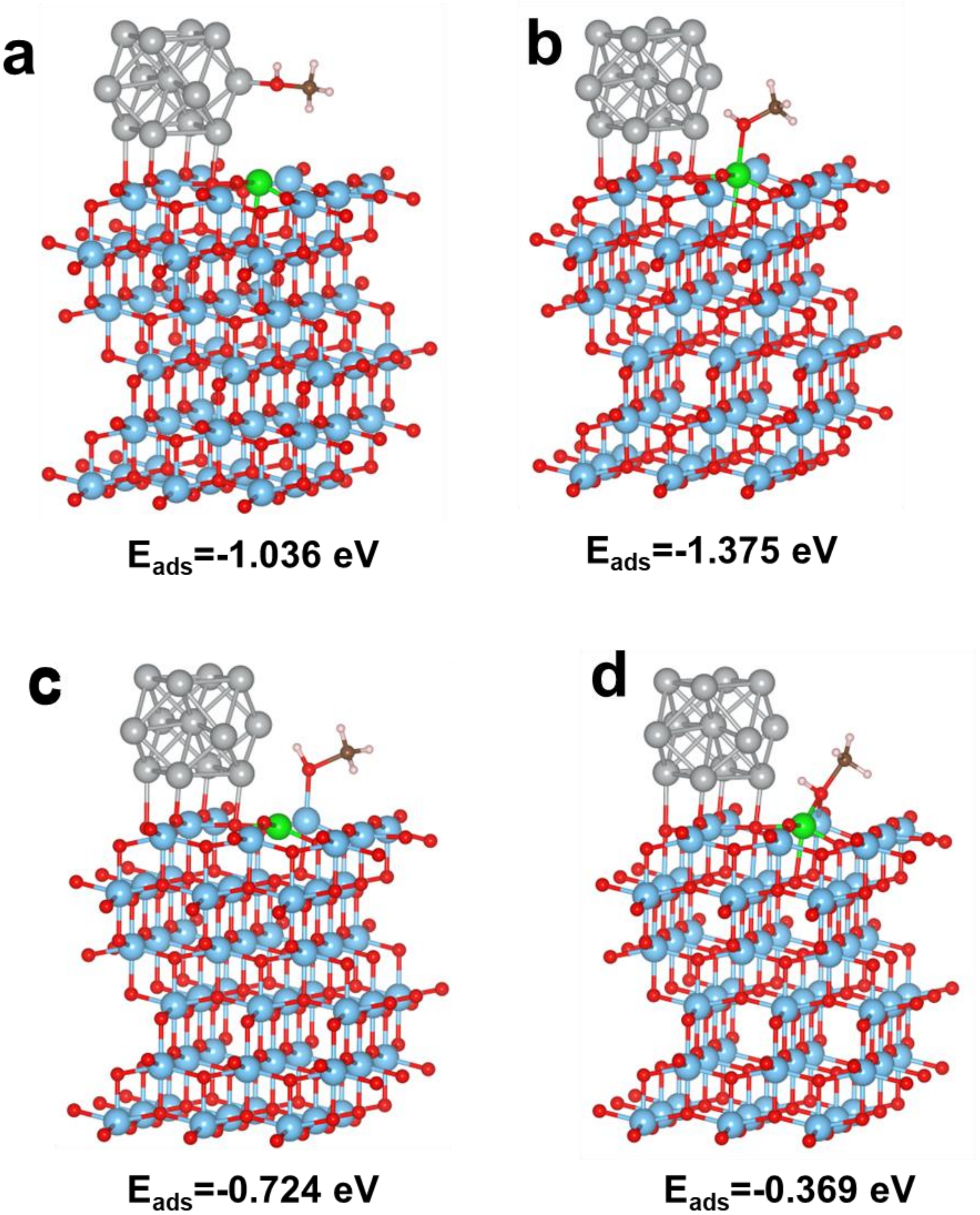

Supplementary Figure 47. Optimized DFT structures for CH_3OH adsorption. **a** Ag, **b** W, **c** Ti, and **d** O_v active sites of Ag.W-BTO, respectively.

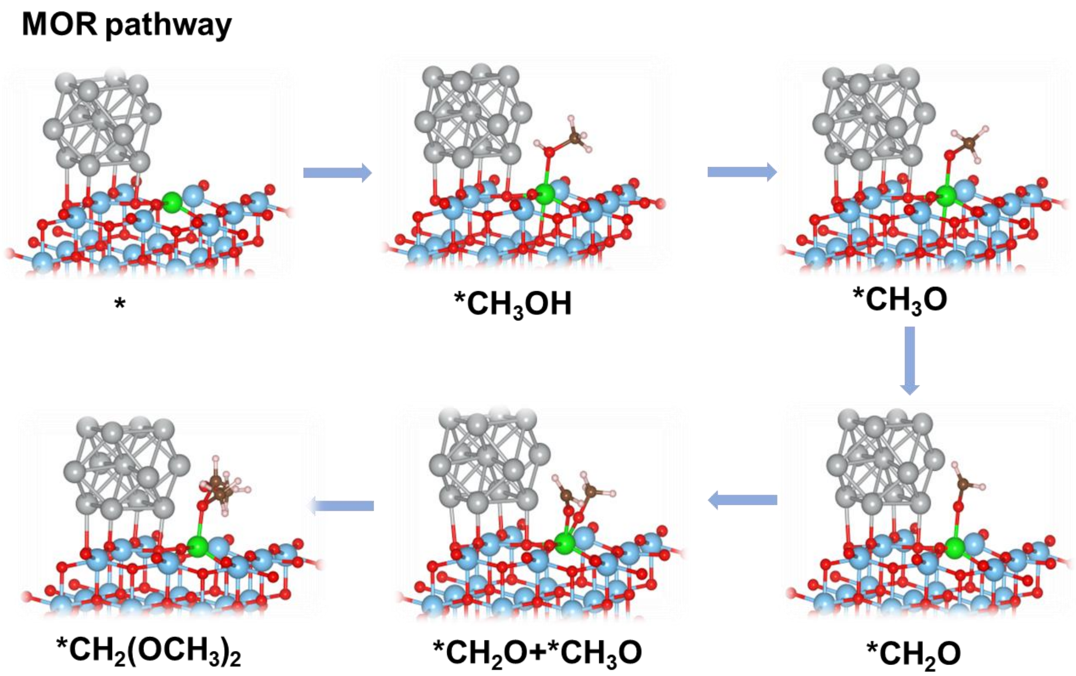

Supplementary Figure 48. Geometries of selected reaction intermediates involved in MOR pathways in the generation of DMM product.

Supplementary Table 1. The chemical contents of different catalysts are determined by XPS.

Samples	Ti (wt.%)	O (wt.%)	W (wt.%)	Ag (wt.%)
BTO	35.642	64.359	0	0
W-BTO	34.841	64.472	0.689	0
Ag-BTO	32.293	65.655	0	2.052
Ag.W-BTO-50	29.395	65.102	2.335	3.168
Ag.W-BTO	30.361	64.578	1.521	3.542
Ag.W-BTO-150	28.967	62.753	2.205	6.085

Supplementary Table 2. Weight% of W and Ag loading obtained by ICP-OES analysis.

Samples	W (wt.%)	Ag (wt.%)
BTO	-	-
W-BTO	19.12	-
Ag-BTO	-	26.74
Ag.W-BTO-50	19.08	18.07
Ag.W-BTO	19.23	28.11
Ag.W-BTO-150	18.91	38.53

Supplementary Table 3. EXAFS fitting parameters at the Ti K-edge for Anatase TiO_2, BTO, Ag-BTO, W-BTO, and Ag.W-BTO

Sample	Bond	CN = N × S_0^2	R (Å)	σ^2 (Å^{-2})	ΔE_0 (eV)	R-factor
**Anatase TiO_2**	Ti–O	6	1.956	0.0091	–1.98	0.0511
	Ti–Ti (1)	4	3.05	0.0089	–9.57	
	Ti–Ti (2)	4	3.842	0.0077	–16.15	
**BTO**	Ti–O	2.4	1.91	0.0025	–9.55	0.0576
**Ag-BTO**	Ti–O	5.4	1.933	0.0083	–4.58	0.0246
	Ti–Ti (1)	1.92	3.01	0.0036	–11.53	
	Ti–Ti (2)	4	3.565	0.0156	4.8	
**W-BTO**	Ti–O	5.22	1.928	0.0081	–5.1	0.0186
	Ti–Ti (1)	1.8	3.003	0.0029	–12.2	
	Ti – Ti (2)	4	3.567	0.0155	4.85	
**Ag.W-BTO**	Ti–O	5.57	1.954	0.0088	–3.6	0.0178
	Ti–Ti (1)	2.2	3.012	0.0040	–13	
	Ti–Ti (2)	4	3.597	0.0143	5.27	

CN: coordination numbers; R: bond distance; σ^2: Debye-Waller factors; ΔE^0: the inner potential correction; R-factor: goodness of fit.

Supplementary Table 4. EXAFS fitting parameters at the Ag K-edge for Ag foil, Ag_2O, Ag-BTO, and Ag.W-BTO

Sample	Bond	CN = N × S_0^2	R (Å)	σ^2 ($Å^{-2}$)	ΔE_0 (eV)	R-factor
**Ag foil**	Ag–Ag	12	2.863	0.0081	–1	0.0007
**Ag_2O**	Ag–O	2	2.062	0.0057	2.12	0.0495
	Ag–Ag	12	3.151	0.0416	–8.08	
**Ag-BTO**	Ag–Ag	10.46	2.860	0.0081	0.63	0.0010
**Ag.W-BTO**	Ag–Ag	8.41	2.857	0.0080	–1.48	0.0022

CN: coordination numbers; R: bond distance; σ^2: Debye-Waller factors; ΔE^0: the inner potential correction; R-factor: goodness of fit.

Supplementary Table 5. EXAFS fitting parameters at the W L_3-edge for W foil, WO_3, W-BTO, and Ag.W-BTO

Sample	Bond	CN = N × S_0^2	R (Å)	σ^2 ($Å^{-2}$)	ΔE_0 (eV)	R-factor
**W foil**	W–W (1)	8	2.756	0.004	7.93	0.0199
	W–W (2)	6	3.177	0.007	9.84	
**WO_3**	W–O (1)	4	1.781	0.0072	1.1	0.0118
	W–O (2)	2	2.097	0.0048	3.04	
**W-BTO**	W–O (1)	3.06	1.887	0.0034	14.9	0.0391
	W–O (2)	2.77	2.245	0.0054	15.69	
**Ag.W-BTO**	W–O (1)	3.88	1.77	0.009	–8.48	0.047
	W–O (2)	1	2.098	0.0005	–1.47	

CN: coordination numbers; R: bond distance; σ^2: Debye-Waller factors; ΔE^0: the inner potential correction; R-factor: goodness of fit.

Supplementary Table 6. The BET, pore size, and pore volume of BTO, W-BTO, Ag-BTO, and Ag.W-BTO.

Samples	BET (m^2 g^{-1})	Pore diameter (nm)	Pore volume (cm^3 g^{-1})
BTO	28.62	29.80	0.25
W-BTO	44.68	40.66	0.36
Ag-BTO	35.26	40.80	0.32
Ag.W-BTO	52.12	32.78	0.40

Supplementary Table 7. Decay lifetimes of fs-TA spectra and their relative percentages of photoexcited charge carriers in the BTO, Ag-BTO, W-BTO, and Ag.W-BTO.

Decay lifetimes	BTO	Ag-BTO	W-BTO	Ag.W-BTO
τ_1 (ps)	0.10 (55%)	0.10 (56%)	0.10 (89%)	0.10 (19%)
τ_2 (ps)	1.40 (45%)	2.88 (44%)	0.20 (11%)	6.71 (60%)
τ_3 (ps)	-	-	-	172.05 (22%)

Supplementary References

1. Gondal M, et al. Preparation of WO_3/g-C_3N_4 composites and their enhanced photodegradation of contaminants in aqueous solution under visible light irradiation. *Reaction Kinetics, Mechanisms and Catalysis* **114**, 357-367 (2015).

2. Kresse G, Furthmüller J. Efficiency of ab initio total energy calculations for metals and semiconductors using a plane-wave basis set. *Comput Mater Sci* **6**, 15-50 (1996).

3. Kresse G, Furthmüller J. Efficient iterative schemes for ab initio total-energy calculations using a plane-wave basis set. *Phys Rev B* **54**, 11169 (1996).

4. Perdew JP, Burke K, Ernzerhof M. Generalized gradient approximation made simple. *Phys Rev Lett* **77**, 3865-3868 (1996).

5. Kresse G, Joubert D. From ultrasoft pseudopotentials to the projector augmented-wave method. *Phys Rev B* **59**, 1758 (1999).

6. Blöchl PE. Projector augmented-wave method. *Phys Rev B* **50**, 17953 (1994).
